# Matching plus regression adjustment for the estimation of the average treatment effect on survival outcomes: a case study with mosunetuzumab in relapsed/refractory follicular lymphoma

**DOI:** 10.1186/s12874-025-02456-x

**Published:** 2025-02-01

**Authors:** Danilo Di Maio, S. A. Mitchell, S. Batson, E. Keeney, Howard H. Z. Thom

**Affiliations:** 1 F. Hoffmann-La Roche Ltd, Basel, Switzerland; 2 Mtech Access, Bicester, Oxfordshire UK; 3 Clifton Insight, Bristol, UK; 4 University of Bristol, Bristol, UK

**Keywords:** Indirect treatment comparisons, Propensity score matching, Regression adjustment, Doubly robust, Counterfactual, ATE

## Abstract

**Background and objectives:**

The National Institute for Health and Care Excellence (England’s health technology assessment body) recommend the use of the average treatment effect (ATE) as an estimand for economic evaluations. However there is limited literature on methods to estimate the ATE, particularly in the case of survival outcomes. Single-arm trials and real-world data are playing an increasing role in health technology assessments, particularly in oncology/rare diseases, generating a need for new ATE estimation methods. This study aimed to present the adaptation and utility of this methodology for survival outcomes.

**Methods:**

The approach is based on a “doubly robust” method combining matching with regression adjustment (Austin 2020) using a Weibull model (lowest Akaike information criteria [AIC] specification) to estimate counterfactual event times. As a case study, we compared mosunetuzumab versus rituximab/bendamustine, as a proxy for rituximab/chemotherapy, in 3L+ relapsed/refractory follicular lymphoma. Individual patient data for mosunetuzumab (NCT02500407) and a combination of two rituximab/bendamustine 3L+ follicular lymphoma cohorts (NCT02187861/NCT02257567) were used. Endpoints included overall survival (OS) and progression-free survival (PFS). Sensitivity analyses were performed to test robustness to different distributional assumptions (log-normal, log-logistic and exponential) or model specifications (second, third and fourth lowest AIC) for event times.

**Results:**

The case study found improved PFS (hazard ratio [HR] 0.43 [95% confidence interval (CI): 0.13, 0.91]) and OS (HR 0.30 [95% CI: 0.05, 5.28]) for mosunetuzumab. Consistent findings (HR range 0.25–0.47 and 0.21–0.50 with all CIs excluding/including 1 for PFS/OS, respectively) were observed in sensitivity analyses.

**Discussion/conclusions:**

The proposed adaptation expands the range of available approaches for the estimation of the (local) ATE for survival outcomes in health technology assessments using “doubly robust” methods. This approach appeared relatively robust to modelling decisions in our case study.

**Supplementary Information:**

The online version contains supplementary material available at 10.1186/s12874-025-02456-x.

## Background

Follicular lymphoma (FL), a form of low-grade non-Hodgkin lymphoma, has a chronic relapsing disease course with long overall survival (OS) [1, 2]. Patients with FL and a low tumor burden can often be considered for observation only. However, if active treatment is required, obinutuzumab or rituximab in combination with chemotherapy can be administered as initial treatment. After a response to initial therapy, recurrent relapses frequently occur with progressively shorter durations of response to salvage therapy [3, 4]. There is no standard of care for relapsed/refractory (RR) FL [5, 6] but treatment often includes rituximab and chemotherapy (R-chemo), particularly in later lines of therapy [7] because other options are not reimbursed in all markets. New agents for third-line and beyond (3L+) treatment of RR FL, mainly investigated in Phase 1/2 single-arm studies, are emerging [8]. This includes mosunetuzumab, a novel CD20/CD3 bispecific antibody (NCT02500407) [9].

To assess the true causal effects of interventions requires evaluation of the average treatment effect (ATE), i.e. the expected relative effect of a treatment on a randomly selected individual from the population, which can normally be estimated by directly comparing mean outcomes between treated and control groups in randomized controlled trials (RCT) [10–12]. The ATE is also the estimand generally preferred by health technology assessment (HTA) bodies, such as England’s National Institute for Health and Care Excellence (NICE), for economic evaluations of new interventions [13], due to its broader generalizability. Although RCTs represent the gold standard to estimate the ATE, they are not always feasible and, despite treatment effect estimates derived from non-randomized studies being at higher risk of bias [14], evidence from single-arm trials and real-world observational data are increasingly used in HTA [10, 12, 14–16]. This is particularly the case in rare diseases or some oncology indications where conducting an RCT may not be possible, patient recruitment may be difficult, or events may take too long to occur [17–26]. These situations necessitate statistical approaches that control for confounding and estimate the counterfactual outcomes in a target population where every patient has received either treatment to evaluate the average causal effect, following Neyman-Rubin’s causal framework [27, 28].

An increasingly popular methods family in observational research uses two regression models, one for the outcome (sometimes referred to as G-formula or Q-model) [29, 30] and one for treatment exposure (i.e. a propensity score [PS] model) [31–36]. The combination of methods modelling the outcome (often referred to as regression adjustment) [37–39] and the exposure (i.e. propensity score matching or weighting) [40–44] has generally been shown to have superior statistical properties versus individual methods alone, as it can protect against bias from potential misspecification of either model [45]. This is often referred to as “double robustness,” an appealing theoretical property which implies that such methods yield consistent estimators if at least one model is correctly specified [33, 46–49]. However, they may not always have superior performance to single model strategies when both regression models are mis-specified [50–52].

The literature on “doubly robust” methods to estimate the ATE is scarce, particularly for continuous survival outcomes, with few examples of code implementation or application to real case studies. In fact, most studies either focus on the estimation of the average treatment effect on the treated (ATT), i.e. the expected relative effect of a treatment on the individuals who received the treatment, or on outcomes other than survival. There is therefore an increasing need to investigate new statistical methods that minimize the risk of bias and enable estimation of the ATE when individual patient data (IPD) are available to compare survival outcomes between treatments.

Currently available “doubly robust” methods include the augmented inverse probability of treatment weighting (AIPTW) [35, 53–55], empirical likelihood estimation [56] or the combination of inverse probability of treatment weighting (IPTW) with G-computation [56, 57]. A recently published method by Austin et al. (2020) [58] combines nearest neighbor [59] or caliper matching [60] with different regression adjustment models to estimate the ATT for time-to-event outcomes. By combining design- (matching) and outcome-based methods of bias reduction, this approach can also be considered to belong to the “doubly robust” family [33, 34, 36]. Owing to the availability of different matching algorithms, this method offers greater flexibility than the currently investigated alternatives, with potential for customization to different dataset characteristics. Furthermore, unlike other popular approaches that rely on a covariate-adjusted outcome model to control for residual imbalances on relative effect estimates after matching/weighting [61], by adjusting the IPD twice this method can also adjust for residual imbalances in survival curves. This may be beneficial when studying relative effect measures other than the hazard ratio (HR) (e.g. difference in survival rates or in mean survival times), as well as when adjusted survival curves are needed for subsequent modelling. Moreover, methods relying on outcome imputation are beneficial in terms of statistical power compared to other established methods, such as those modelling treatment exposure only, even when censoring rates are high [55, 62, 63]. This may be particularly useful in rare/indolent diseases, such as 3L+ FL, where recruiting patients and/or accruing events may be challenging.

This study was inspired by Austin et al. (2020) [58] and its objective was twofold: (1) propose an adaptation of their reported method for estimation of the ATE and develop a code example (not available in the original article) to facilitate its adoption; (2) illustrate its implementation in a HTA application context, such as the comparison between two treatments using different data sources. For the latter, the comparison of mosunetuzumab and rituximab plus bendamustine (BR; as a representative for R-chemo) for the 3L+ treatment of RR FL was used as a case study.

## Methods

### Data sources

The ATE estimation requires availability of IPD for both treatments, which in real-world applications usually come from clinical trial or registry/claims data. For this study, trial data on OS and progression-free survival (PFS) were available and were preferred as they typically represent a higher quality source than registry data [64], due to generally stricter enrolment and data collection requirements [65, 66]. The source of mosunetuzumab single-agent data was the dose expansion cohort of GO29781 (NCT02500407) [9], which included 90 patients with 3L+ RR FL, based on a clinical cut-off date of January 2022. The population used to compare mosunetuzumab to BR was a combination of two cohorts of patients with 3L+ FL from the BR arms of the CONTRALTO (NCT01724021) [67] and GO29365 (NCT02257567) [68] trials (*n* = 48, latest available cut-off date for both). There were important differences in follow-up between treatment arms. Although this was not unexpected, as CONTRALTO and GO29365 are completed studies whereas GO29781 is still ongoing, this likely had an impact on the difference in censoring rates across arms, particularly given the slowly progressive nature of FL [2].

To ensure that the patient cohorts used for the analyses were as homogeneous as possible, a filtering procedure based on applying common eligibility/enrolment criteria was adopted. This consisted of excluding patients with Eastern Cooperative Oncology Group Performance Status (ECOG PS) 2 from the CONTRALTO and the GO29365 trial cohorts (to align with the inclusion/exclusion criteria of the GO29781 trial) and excluding patients who received more than five prior anticancer regimens from the GO29781 cohort (to align with the populations enrolled in the CONTRALTO and GO29365 trials). This allowed improvement of population overlap prior to conducting any adjustments. In total, 81 and 46 patients were included in the mosunetuzumab and BR arms, respectively. In the case of missing values for categorical or continuous covariates, these were imputed using a simple imputation approach based on the mode or the mean without the missing values, respectively. This was justified by the limited amount of missing data observed across datasets, due to these originating from clinical trials.

### Statistical approach

#### Propensity score estimation

The propensity score $\:{e}_{i}=\text{Pr}\left({z}_{i\:}=1\:\right|{\varvec{x}}_{i\:})$ is estimated using the following logistic model:

$$\:\text{log}\left(\frac{{e}_{i}}{{1-e}_{i}}\right)={\beta\:}_{0}+\:{\varvec{\beta\:}\varvec{x}}_{i}$$

Where $\:{z}_{i\:}$is the treatment exposure indicator variable, $\:{\varvec{x}}_{i\:}$a vector or relevant covariates for individual $\:i$, $\:{\beta\:}_{0}$ a model intercept and $\:\varvec{\beta\:}$ a vector of model coefficients for the parameters $\:{\varvec{x}}_{i\:}$. The pre-specified list of prognostic factors and effect modifiers considered for the analysis, ranked by level of priority, was informed by clinical expert opinion (Table 1). Expert elicitation was obtained via direct discussions with independent clinical experts. Only variables that could influence outcomes (and potentially treatment exposure) were selected for inclusion in the models; variables that would be associated with exposure only (e.g. instrumental variables) were excluded, to avoid introducing bias in the analyses. The inclusion of selected two-way covariate interaction terms with potential prognostic value (i.e. age and number of prior therapies, age and refractory to last line of therapy and progression of disease within 24 months [POD24] and refractory to last line of therapy), as per consultation with clinical experts, was also considered.

**Table 1:** Prognostic factors and/or effect modifiers considered for inclusion in the analysis, ranked by level of priority^ǂ^

Prognostic factor /effect modifier	Direction of effect with increasing covariate values*	Notes
***High priority***		
Number of previous chemotherapeutic (or systemic) agents	↓	E.g., 3 vs. >3 [no clinically established threshold] or median, if categories not reported
Refractoriness to last previous therapy (yes/no)	↓	E.g. defined as best response progressed/stable disease or relapse < 6 months from last therapy
Refractoriness to any prior aCD20 mAb containing therapy (yes/no)	↓	Also used as proxy for rituximab refractoriness when needed
Early relapse status (POD24) (yes/no)	↓	Can be defined differently across studies; definition: “from first treatment” should be preferred over “from diagnosis”, as it is aligned with the definition used in the GO29781 trial
Prior (A)SCT (yes/no)	↓	
Size of the largest lymph node lesion (longest dimension) involved	↓	Highly correlated with bulky disease^1^ -> to be prioritized over bulky disease, when possible
Bulky disease (yes/no)^a^	↓	
Follicular lymphoma international prognostic index risk group (high [≥ 3] versus intermediate/low [< 3])	↓	At study entry; if at diagnosis, limited value in 3 L + and individual components to be prioritized
Age	↓	Mean, or median if mean not reported, or % ≥60 years (when feasible), if neither reported
Ann Arbor Stage (III–IV vs. I-II)	↓	
High lactate dehydrogenase (yes/no)	↓	
Bone marrow involvement (yes/no, as demonstrated by bone marrow biopsy)	↓	When available, typically is rarely reported
Low hemoglobin level (yes/no, e.g. <12 [or 12.5] g/dL, or < LLN)	↓	
	***Low priority***	
Duration of prior response/time in previous remission/time since completion of last therapy	↑	
Presence of B-symptoms (not that important as they are quite subjective)	↓	
ECOG S (e.g. 1 vs. 0)	↓	The distinction between 0–1 and ≥ 2 carries the greatest prognostic value

Abbreviations: (A)SCT, autologous stem cell transplant; ECOG PS, Eastern Cooperative Oncology Group performance status; LLN, lower limit of normal. *The arrows indicate the impact on survival times (↓ decreases, ↑ increases), i.e. ↓ is associated with higher prognostic impact. ǂInformed by clinical expert opinion

^a^Bulky disease is generally constructed from the size of largest lymph node lesion (longest dimension) involved; as none of the thresholds typically used to define bulky disease have been established as being superior prognostically over the others (based on medical feedback), then adjusting for bulky disease in the analysis should be de-prioritized in favour of size of largest lymph node lesion when information on both is available. It is worth noting that high tumor burden/bulky disease based on GELF criteria can also be reported in some studies. However, this is mainly used to decide between a watch-and-wait and an active treatment approach and its prognostic value is not really recognized by the medical community

If the coefficients or standard errors (SEs) of the logistic regression model used to estimate the PSs were implausibly large (e.g. over 1,000), indicating excessive treatment group heterogeneity or collinearity issues, the problematic covariate(s) was identified and excluded from the model (eventually not required). The final distribution of estimated PSs was inspected in order to assess the overlap between treatment groups, as well as to identify the presence and frequency of PSs with potentially extreme values, which could create problems during the matching process (Figure S1).

#### Propensity score matching

Five matching methods were explored: (1) nearest neighbor matching without replacement [59]; (2) nearest neighbor matching with replacement [59]; (3) optimal pair matching [69]; (4) genetic matching [70]; and (5) full matching [71, 72]. The matching method and PS model that resulted in the optimal covariance balance, i.e. that which minimized the number of imbalanced confounders (overall and within the high priority set), was selected as the preferred approach. Effective sample sizes (ESS) were also compared across methods to ensure the bias-variance trade-off was acceptable.

#### Assessment of covariate balance

Covariate balance was assessed as measured by the absolute standardized mean differences, using a threshold of 0.1, and the complements of overlapping coefficients pre- and post-matching [73, 74].

#### Regression adjustment

Regression adjustment was conducted on the matched patient sample in order to estimate the counterfactual outcomes (i.e. missing potential outcomes under treatment for controls and missing potential outcomes under control for treated subjects) required to recover the ATE or a local form of it (i.e. the ATE on a subsample of individuals from the overall population). In fact, the matching method selection may have implications on the interpretation and generalizability of the results, as using a less representative sample may result in a change to the estimand being evaluated [59].

Unlike Austin et al. (2020) who only imputed potential outcomes under control for each treated patient (as the aim was to estimate the ATT), all counterfactual outcomes were imputed in this case, as the aim was to recover the ATE [27, 28, 63, 75–77]. To achieve this, an accelerated failure time (AFT) parametric model was used to regress event times on baseline characteristics:

$$\:\text{log}\left({T}_{i}\right)={\beta\:}_{0}+{\varvec{\beta\:}\varvec{x}}_{i}+\sigma\:{\epsilon\:}_{i}$$

Where $\:{T}_{i}$ is the event time for individual $\:i$, $\:\sigma\:$ a scale parameter, $\:{\epsilon\:}_{i}\:$the error terms and $\:{\beta\:}_{0}$, $\:\varvec{\beta\:}$ and $\:{\varvec{x}}_{i}$ the model intercept, coefficient and covariate values, respectively, as per the notation above.

This modelling approach was selected as it provides additional flexibility and is more straightforward to implement in a code compared with other potential options such as semi-parametric Cox regression models, which require additional steps (i.e. estimation of a baseline hazard and subsequent sampling of event times) [58]. Moreover, results from a recent simulation study showed that most methods relying on Cox models may have systematic bias in small samples, even when the model is correctly specified [76], indicating that an alternative model may be more appropriate given the sample size of our case study.

A Weibull model was selected as the reference based on its advantageous theoretical properties, e.g. as it was the only model which simultaneously fulfilled both the proportional hazards (PH) and accelerated failure time assumptions, providing sufficient flexibility to capture the relationship between patient characteristics and outcomes under multiple possible scenarios [78]. It is worth nothing that other parametric models may have also been potentially suitable candidates; therefore, the impact of selecting alternative models was assessed in scenario analyses.

Although outcome models should preferentially include all risk factors, the overall small sample size in our dataset, as well as the relatively short follow-up in the treated group and the limited number of events observed for some endpoints, warranted the use of a reduced set of covariates, as also highlighted in previous studies [58, 62]. Similarly, two-way interactions were not considered because of the large number of potential factors for inclusion. Therefore, we aimed to select an optimal model specification that would allow us to best capture the confounder-outcome relationship as observed in the available data. The selection of covariates in the final regression model was performed by comparing the Akaike information criteria (AIC), a score used to determine model performance with a penalty for the number of model parameters, of all combinations of the available covariates [79, 80]. Excluding the interactions enabled all models to be initially considered for minimum AIC exploration and minimized the potential for overfitting. Different outcome models for the treated and the control groups were allowed and event times estimated to be larger than the maximum follow-up between the treatment groups being compared were censored to avoid extrapolating excessively beyond the range of observed data. Covariates that made the final model unstable (e.g. leading to at least one coefficient SE being excessively high or 0) and prevented it from converging properly (e.g. if no event was observed for one category) were removed from the starting set. In line with the original Austin et al. (2020) method, only a single event time per-patient was imputed, for simplicity (the imputation of multiple event times would have required the additional step of using Rubin’s rules to obtain a pooled estimate of the HR SEs) [81].

The counterfactual outcomes for each group estimated as described above were combined with the respective observed outcomes. The resulting dataset was then used to compare OS and PFS outcomes between treatments using standard survival analysis methods (i.e. Kaplan-Meier (KM) estimators and Cox regression models).

Bootstrapping over the entire matching and regression adjustment procedure, which has the advantage of yielding valid post-selection inference, was used to obtain confidence intervals (CI) (2000 resamples were used to ensure a minimum of 1000 values for the estimation) [82].

A series of scenario analyses were conducted for the regression adjustment approach to test its robustness to different modeling decisions, such as the use of different distributional assumptions and statistical models for event times. The scenarios explored included the use of alternative AFT parametric survival models for the distribution of event times (Weibull versus log-normal, log-logistic, and exponential). Together, these represent the four most frequently accepted distributions in HTA applications [83, 84]. Note that other distributions among those commonly considered in HTA [85], such as Gompertz, Generalized Gamma, or other more flexible parametric models, could not be explored as these would either (1) likely be implausible for the case study (by modelling “curative-like” scenarios [86, 87] in the context of a chronic relapsing disease considered incurable [7]), (2) be excessively data demanding, or (3) not be supported by the package used to simulate event times. Also, alternative outcome regression model specifications were explored (lowest AIC versus second, third and fourth-lowest AIC).

#### Software

All analyses were performed using the R statistical programming language [88]. Model selection by stepwise backward-testing was conducted using the stepAIC function [79]. The ‘MatchIt’ package was used to estimate the propensity score and for matching [89]. Covariate balance was assessed using the cobalt package [90]. Survival analysis was conducted and plots were created using the R packages ‘survival’ and ‘survminer’ [91]. Simulation of survival outcomes for the regression adjustment was performed using the R package ‘survParamSim’ [92]. Restricted mean survival times (RMST) were estimated using the survRM2 R package (at the shortest maximum follow-up across arms, as per default option). The analysis R code is provided in the supplementary information.

## Results

### Model and matching algorithm selection

A final model including all linear predictors plus selected clinically relevant interaction terms (see Methods), was selected to estimate the propensity score, as it yielded better covariate balance compared to a model without interactions (Table S1 & Figure S2). Covariate balance achieved with the different matching methods is displayed in love plots in Figure S3. The final sample sizes after nearest neighbor matching without replacement, genetic matching and optimal pair matching were 92 patients (as no weights are involved). The final ESS after nearest neighbor matching with replacement was 81 patients in the mosunetuzumab arm and ~ 9.9 pseudo-patients in the BR arm. The final ESS after full matching (ATE) was ~ 49.3 pseudo-patients in the mosunetuzumab arm and ~ 18.9 pseudo-patients in the BR arm. Balance after nearest neighbor matching without replacement and genetic matching was quite poor on several covariates and nearly indistinguishable across the two methods. Balance after full matching (ATE) and nearest neighbor matching with replacement was also fairly poor. Optimal pair matching instead resulted in a better, though still not fully achieved, covariate balance compared with all other matching methods explored. Because of this, optimal pair matching was selected as the matching method of preference for the comparison. Although covariate balance post-matching was improved significantly for many prognostic factors (Table 2), residual imbalances for several factors were observed, most of which were, notably, in favor of the BR arm from a prognostic perspective. This is not necessarily unexpected, given the small sample size of the BR arm, as well as the fact that the CONTRALTO and GO29365 trials (being clinical studies focusing on the broader RR population) enrolled comparatively healthier patients (i.e. less refractory and less heavily pre-treated) vs. GO29781, which instead prioritized enrolment of patients with no other suitable treatment options.

**Table 2:** Summary of baseline characteristics after optimal pair matching

Variable	Mosunetuzumab (SS = 46)		Rituximab plus bendamustine (SS = 46)		aSMD	Complement of the overlapping coefficient
	Mean	SD	Mean	SD		
Age (mean)	62.7	12.2	63.3	11.2	0.04	0.15
ECOG PS (1 vs. 0) (%)	32.6	46.9	26.1	43.9	0.13	0.07
FLIPI ≥ 3 (Yes) (%)	47.8	50.0	50.0	50.0	0.04	0.02
Ann Arbor Stage III/IV (Yes) (%)	73.9	43.9	80.4	39.7	0.15	0.07
Prior therapies ≥ 3 (%)	63.0	48.3	60.9	48.8	0.04	0.02
Refractory to last line (Yes) (%)	60.9	48.8	47.8	50.0	0.28	0.13
Refractory to any prior anti-CD20 mAb containing regimen (Yes) (%)	76.1	42.7	69.6	46.0	0.15	0.07
Double refractory (Yes) (%)	47.8	50.0	45.7	49.8	0.04	0.02
POD24 (Yes) (%)	52.2	50.0	47.8	50.0	0.09	0.04
Bone marrow involvement (yes) (%)	28.3	45.0	26.1	43.9	0.05	0.02
Prior ASCT (Yes) (%)	13.0	33.7	6.5	24.7	0.16	0.07
Size of the largest node lesion [cm] (mean)	5.4	2.6	5.7	3.3	0.13	0.09
Low Hgb (Yes) (%)	30.4	46.0	30.4	46.0	0.00	0.00
High LDH (Yes) (%)	34.8	47.6	32.6	46.9	0.04	0.02
Time since completion of last therapy [months] (mean)	16.3	19.9	26.0	31.9	0.57	0.12

Abbreviations: ASCT, autologous stem cell transplant; aSMD, absolute standardised mean difference; ECOG PS, Eastern Cooperative Oncology Group Performance Status; FLIPI, Follicular lymphoma international prognostic index; Hgb, hemoglobin; LDH, lactate dehydrogenase; POD24, Progression of disease within 24 months; SS, sample size. Balanced (defined as < 0.1 for aSMD)

Regression parameters for all final outcome models explored are reported in Table S2. The covariates that had to be removed from the starting set for each treatment and outcome model are reported in the R code available in the supplementary information. The main reason for model instability or convergence issues was the lack of observed events for one category of a binary covariate. It is worth noting that the direction of the coefficients in the final models was generally in line with the anticipated prognostic impact of the variables included.

### Indirect comparison results

A summary of the OS and PFS HR and RMST difference outcomes for mosunetuzumab versus BR assessed using the matching plus regression adjustment approach is provided in Tables 3 and 4, respectively. Results from unadjusted and matched (with subsequent covariate adjustment for residual imbalances in the post-matching outcome model) [62] samples are presented in Tables S3 and S4, respectively, in the supplementary appendix, which also includes additional RMST and survival estimates for the matching plus regression adjustment scenarios explored in Table S5. There were extremely high levels of uncertainty associated with the HR and RMST difference estimates in the analyses of OS, which was anticipated due to the low number of events observed in the datasets for both treatment arms.

**Table 3:** Summary of HR matching plus regression adjustment results for mosunetuzumab versus BR

Method for estimating HR	HR (95% CI)	
	OS	PFS
Reference case (lowest AIC and assuming Weibull distribution of event times)	0.30 (0.05, 5.28)	0.43 (0.13, 0.91)
Regression adjustment (second lowest AIC model* and assuming Weibull distribution of event times)	0.41 (0.06, 6.47)	0.44 (0.13, 0.97)
Regression adjustment (third lowest AIC model* and assuming Weibull distribution of event times))	0.21 (0.05, 5.67)	0.45 (0.11, 0.96)
Regression adjustment (fourth lowest AIC model* and assuming Weibull distribution of event times))	0.39 (0.07, 5.65)	0.42 (0.13, 0.95)
Regression adjustment (lowest AIC, assuming log-normal distribution of event times)	0.27 (0.04, 1.83)	0.28 (0.11, 0.88)
Regression adjustment (lowest AIC, assuming log-logistic distribution of event times)	0.29 (0.06, 2.46)	0.25 (0.11, 0.93)
Regression adjustment (lowest AIC, assuming exponential distribution of event times)	0.50 (0.07, 4.16)	0.47 (0.17, 0.87)

Abbreviations: AIC, Akaike information criterion; BR, rituximab plus bendamustine; CI, confidence interval; HR, hazard ratio; OS, overall survival

HRs presented for the comparison of mosunetuzumab versus rituximab plus bendamustine. HRs < 1 favor mosunetuzumab

*Without convergence issues

**Table 4:** Summary of RMST difference matching plus regression adjustment results for mosunetuzumab versus BR

Method for estimating RMST difference	RMST difference (95% CI)	
	OS	PFS
Reference case (lowest AIC and assuming Weibull distribution of event times)	5.00 (-5.40, 11.56)	7.57 (-0.29, 14.70)
Regression adjustment (second lowest AIC model* and assuming Weibull distribution of event times)	4.46 (-5.99, 11.12)	7.59 (-0.55, 14.96)
Regression adjustment (third lowest AIC model* and assuming Weibull distribution of event times))	5.82 (-6.06, 11.49)	6.54 (-1.00, 15.25)
Regression adjustment (fourth lowest AIC model* and assuming Weibull distribution of event times))	4.03 (-5.58, 9.13)	6.88 (-0.76, 14.38)
Regression adjustment (lowest AIC, assuming log-normal distribution of event times)	4.15 (-1.93, 11.95)	9.34 (-1.01, 15.52)
Regression adjustment (lowest AIC, assuming log-logistic distribution of event times)	3.29 (-3.14, 10.28)	9.57 (-0.67, 15.39)
Regression adjustment (lowest AIC, assuming exponential distribution of event times)	2.20 (-15.19, 7.17)	6.47 (-0.62, 13.51)

Abbreviations: AIC, Akaike information criterion; BR, rituximab plus bendamustine; CI, confidence interval; OS, overall survival; RMST, restricted mean survival time

RMST differences presented for the comparison of mosunetuzumab versus rituximab plus bendamustine. RMST difference > 1 favor mosunetuzumab

*Without convergence issues

### Overall survival

**Fig. 1:**
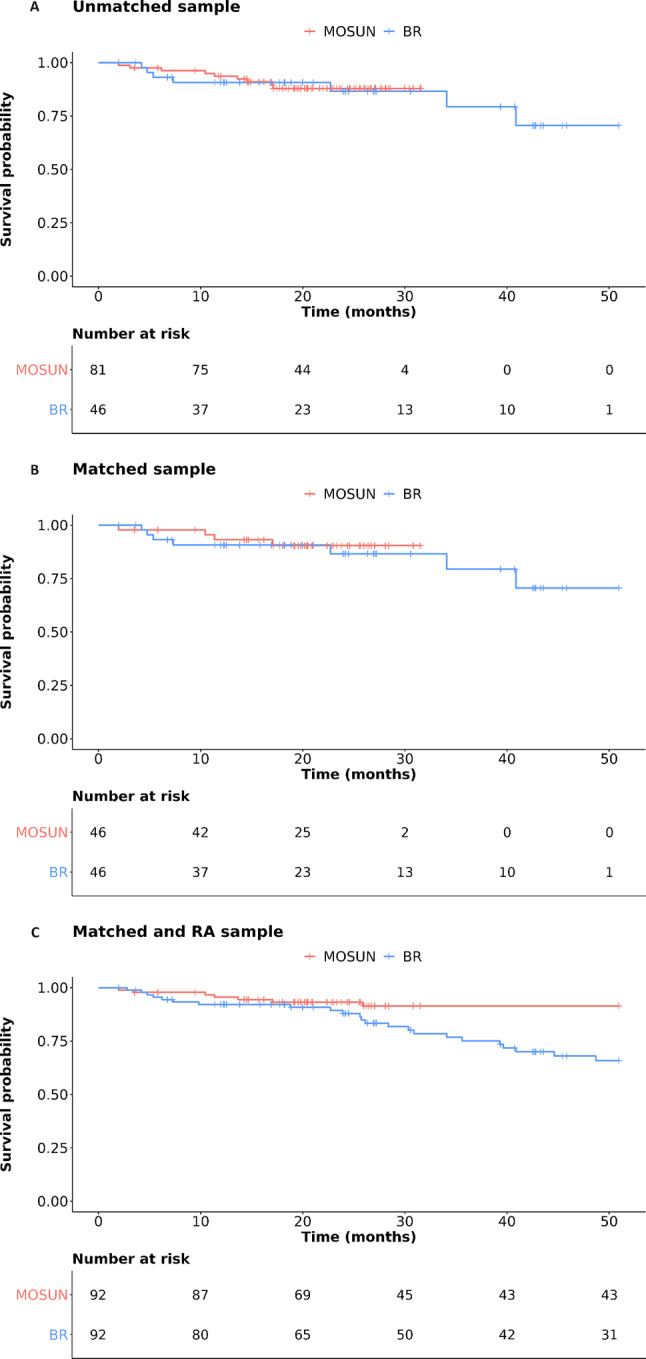
KM plots of OS for unadjusted (**A**), optimal pair matched (**B**) and matching plus regression adjustment samples (**C**)

The OS KM plots for the unadjusted and optimal-pair matching analyses, as well as those generated using the reference matching plus regression adjustment approach are presented in Fig. 1. The KM plots for all the other modelling scenarios explored are presented in Figure S4. The HRs for OS numerically favored mosunetuzumab versus BR (HR < 1) in all analyses using the matching plus regression adjustment method (Table 3). The HR (95% CI) obtained using the reference matching plus regression adjustment model was 0.30 (0.05, 5.28). More favorable (lower) HRs were obtained for the regression adjustments assuming log-normal distribution of event times (0.27 [0.04, 1.83]), assuming log-logistic distribution of event times (0.29 [0.06, 2.46]), and using the third lowest AIC model without convergence issues (0.21 [0.05, 5.67]). Less favorable (higher) HRs were obtained for the regression adjustments using the second lowest AIC model without convergence issues (0.41 [0.06, 6.47]), using the fourth lowest AIC model without convergence issues (0.39 [0.07, 5.65]), and assuming exponential distribution of event times (0.50 [0.07, 4.16]). The RMST difference for OS in the reference matching plus regression adjustment model was 5.00 (-5.40, 11.56) months with the point estimates ranging from 2.20 to 5.82 months across sensitivity analyses. The RMST difference results for OS were overall concordant with the HR results, in that they numerically favored or slightly favored mosunetuzumab versus BR (RMST difference > 1) in all analyses, but featured wide CIs which systematically crossed zero, therefore indicating large uncertainty around the estimated differences.

### Progression-free survival

**Fig. 2:**
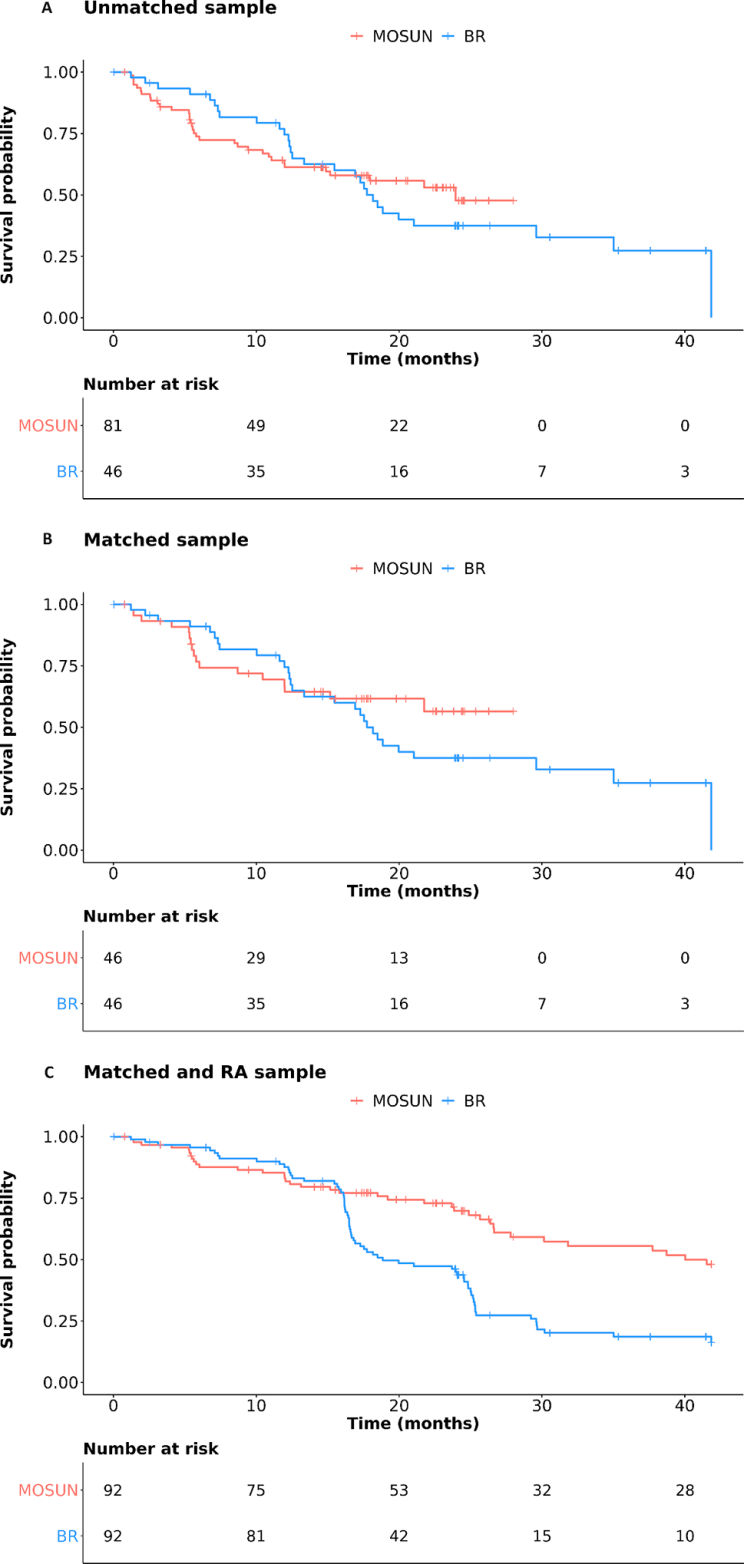
KM plots of PFS for unadjusted (**A**), optimal pair matched (**B**) and matching plus regression adjustment samples (**C**)

The PFS KM plots for the unadjusted and optimal-pair matching analyses, as well as those generated using the reference matching plus regression adjustment approach, are presented in Fig. 2. The KM plots for all other scenarios are presented in Figure S4. The HRs for PFS were < 1 in all analyses using the matching plus regression adjustment method, showing a greater numerical benefit in PFS for mosunetuzumab compared to BR (Table 3). A HR (95% CI) of 0.43 (0.13, 0.91) was obtained using the reference matching plus regression adjustment model. The adjustments assuming log-logistic distribution of event times, log-normal distribution of event times, and using the fourth lowest AIC model without convergence issues had more favorable (lower) HR (95% CI) values of 0.25 (0.11, 0.93), 0.28 (0.11, 0.88), and 0.42 (0.13, 0.95), respectively, compared with the reference matching plus regression adjustment model. In contrast, slightly less favorable (higher) HRs (95% CI) were obtained with the adjustments using the second (0.44 [0.13, 0.97]) and third lowest AIC model without convergence issues (0.45 [0.11, 0.96]), as well as the method assuming exponential distribution of event times (0.47 [0.17, 0.87]). The CIs associated with the HRs did not cross 1 for any of the matching plus regression adjustment scenarios considered. The RMST difference for PFS in the reference matching plus regression adjustment model was 7.57 (‑0.29, 14.70) months with the point estimates ranging from 6.47 to 9.57 months across sensitivity analyses. The RMST difference results for PFS were overall broadly concordant with the HR results, in that they numerically strongly favored mosunetuzumab versus BR (RMST difference > 1) in all analyses, although the respective CIs around the estimated differences marginally crossed 1 in all scenarios considered.

The survival curves (easier to interpret in this case due to the higher number of events) for mosunetuzumab were fairly similar, regardless of whether Weibull or exponential distributions were assumed. However, these differed from those assuming log-normal or log-logistic distributions, which in turn were fairly similar to each other and showed a trend towards a plateau. This may reflect the intrinsic distributional features of these models, as it was not evident in the curves generated using the Weibull model with the fourth lowest AIC, which shared the same specification. This is also in line with what was observed for the BR group, where the survival curves for log-normal or log-logistic distributions had a more similar shape than those obtained assuming exponential or Weibull distributions (third lowest AIC), when all models shared the same specification.

## Discussion

The objective of this study was to adapt a matching plus regression adjustment approach for the estimation of the ATE for survival outcomes, as well as to test the robustness of the method to different modelling decisions through a case study in a rare disease setting. We did not intend to provide an overview of available methods for causal inference of survival outcomes (refer to Denz et al. [2023] [76] for a recent review), nor to compare its performance with that of other methods, as this warrants a thorough simulation study under multiple different data generating assumptions.

In addition to focusing on the ATE, our adaptation differs from the method proposed by Austin et al. (2020) in several ways. While outcome models were fitted separately to both matched patient groups to improve population overlap and reduce the amount of extrapolation beyond observed data when estimating the counterfactual outcomes, we allowed variability between outcome models in the final selection of covariates. Although Austin et al. (2020) did not explore this possibility, presumably due to the larger sample sizes used in their simulation and case study, we consider this an appropriate strategy in applications involving sparse datasets. By allowing more flexibility and not forcing the regression models to share the same specification, there is no need to assume that the covariate-outcome relationship in both treatment groups is best captured using a common model structure, which may not hold true when sample sizes and/or number of events are limited or data maturity differs across arms. We used an AIC-based approach for the final model selection; more sophisticated machine learning methods [93–95] were not considered for simplicity, although they represent valuable strategies for when less sparse datasets are available and interaction terms can be included. Moreover, we censored event times estimated to be larger than the maximum follow-up between treatment groups. Although this differs from the Austin et al. (2020) method, their simulation study did not have imbalances in follow-up across treatments and their real-world case study dataset was close to full data maturity. This represents a somewhat idealized condition, which is often not encountered in HTA (e.g. in external control studies) [16, 96]. We thus consider this approach to be appropriate for our case and similar data maturity situations, particularly when imperfect matching (i.e. one patient group has an overall poorer health status) is an issue, as this may exacerbate the risk that some predicted event times fall excessively beyond the range of observed data. With imperfect matching, using stricter censoring thresholds may cause the undue censoring of informative event times, e.g. if these were predicted for patients in better health had they been part of the sicker group. In fact, when the matched arm with better health has longer follow-up, the counterfactual times for this arm are more likely to be censored compared to those for the other arm, creating an imbalance. However, with more comparable follow-up between arms (e.g. when comparing treatments within the same data source) and greater data maturity, avoiding censoring event times may be useful, as it may help detect if a regression model has major misspecification issues.

Two main findings can be drawn from our case study. Firstly, despite variability in the HRs, the conclusions of our comparison were robust to variation of different assumptions and model specifications used for the outcome regressions. The observed variability is not surprising [97], as our matched sample featured residual imbalances and we could not include all potentially relevant covariates in the outcome models. Nevertheless, all results showed a greater separation between the survival curves, in line with the difference in prognostic variables between groups post-matching, as well as the increase in sample size compared to the original matched sample. These consistently indicated longer PFS (particularly after 16–20 months) and hinted toward a potential numerical OS benefit with mosunetuzumab; however, this remains extremely uncertain, which is to be expected for this indication and follow-up, cautioning against overinterpreting the trends observed in the analyses. Although imputing multiple counterfactual outcomes may have helped increase precision and reduce uncertainty [98], it would not have fundamentally altered the analysis conclusions given the estimated PFS/OS HR CIs [98], so it was not considered. Secondly, our scenario analyses suggested that the selection of the event time parametric distribution model may have an impact on the ensuing survival estimates (as expected from a method explicitly modeling outcomes) [58], particularly in cases where distributions may differ by treatment. However, in practical applications the “correct” event time distribution is rarely, if ever, known. A potential approach to inform the parametric distribution prioritization may rely on fitting different models by treatment on the matched sample and assessing the best visual/statistical fit to the data, potentially followed by clinical validation, as per standard practice in cost-effectiveness modelling [85]. When using these estimates to inform economic models, especially those driven by survival curves, this would also help reduce the uncertainty in cost-effectiveness results, and ultimately decision-making, due to the variability between different modeling scenarios. On the other hand, by using the same distributional assumption for both treatments and combining predicted with observed outcomes, the overall bias may be limited and potentially cancelled out, especially when a common survival distribution can be reasonably assumed.

Our study has limitations. Firstly, the deliberate selection of a challenging and sparse dataset for our HTA case study may limit the generalizability of these findings to other contexts, e.g. less marked and/or more heterogeneous treatment effect differences. Secondly, we were unable to examine how different our “local” ATE results would be from the ATE estimates, as the small control group ESS yielded by full matching prevented us from reliably estimating them. Finally, the clinical disease course and the overall small sample size and sparse nature of the dataset, as well as the current implementation of the survParamSim package, limited the parametric distribution functions of event times we had available to test and the use of Cox regression to estimate survival times was not tested. However, assuming that the time-to-event generating process can be described using a Cox PH model is often an oversimplification [76], which frequently does not hold in HTA applications [99], despite reflecting assumptions made by most researchers.

## Conclusions

In summary, our case study findings were relatively robust to modelling decisions and the proposed adaptation may represent a suitable indirect comparison approach for estimation of the (local) ATE for survival outcomes, offering important benefits in HTA applications. By allowing estimation of “doubly adjusted” (and thus theoretically less biased) survival curves, it may be used to subsequently perform parametric survival extrapolation over a lifetime horizon (a frequent requirement in cost-effectiveness analyses), potentially yielding more robust long-term survival estimates and reducing the uncertainty in the incremental cost-effectiveness ratio. This may be especially beneficial when the PH assumption is unlikely to hold and independent modelling of survival curves between treatments is warranted. However, consideration should be given to the selection of the most appropriate parametric model(s), as the event time distributional assumptions used may have an impact on survival estimates and ultimately cost-effectiveness results.

## Electronic supplementary material

Below is the link to the electronic supplementary material.

Supplementary Material 1

## Data Availability

For eligible studies qualified researchers may request access to individual patient-level clinical data through a data request platform. At the time of writing this request platform is Vivli, at https://vivli.org/ourmember/roche/. For up-to-date details on Roche’s Global Policy on the Sharing of Clinical Information and how to request access to related clinical study documents, see https://go.roche.com/data_sharing.

## Abbreviations

3L+: Third-line and beyond
AFT: Accelerated failure time
AIC: Akaike information criteria
AIPTW: Augmented inverse probability of treatment weighting
ATE: Average treatment effect
ATT: Average treatment effect on the treated
BR: Rituximab plus bendamustine
CI: Confidence intervals
ECOG PS: Eastern Cooperative Oncology Group Performance Status
ESS: Effective sample sizes
FL: Follicular lymphoma
HR: Hazard ratio
HTA: Health technology assessment
IPD: Individual patient data
IPTW: Inverse probability of treatment weighting
KM: Kaplan-Meier
NICE: National Institute for Health and Care Excellence
OS: Overall survival
PFS: Progression-free survival
POD24: Progression of disease within 24 months
PH: Proportional hazards
PS: Propensity score
R-chemo: Rituximab and chemotherapy
RCT: Randomized controlled trials
RMST: Restricted mean survival time
RR: Relapsed refractory
SE: Standard errors
