# Supplementary material for: Matching plus regression adjustment for the estimation of the average treatment effect on survival outcomes: a case study with mosunetuzumab in relapsed/refractory follicular lymphoma
Source: BMC Med Res Methodol. 2025 Feb 1;25:30. doi: 10.1186/s12874-025-02456-x (PMC11786573; doi:10.1186/s12874-025-02456-x)
Supplement: Supplementary file 1 — Supplementary Material 1 [file 12874_2025_2456_MOESM1_ESM.docx]

## Supporting information

## Tables

Table S1: Summary of propensity score model parameters and fit statistics

| Variable | Estimate | SE | p-value |
| --- | --- | --- | --- |
| (Intercept) | 0.14 | 2.38 | 0.95 |
| Age (mean) | 0.00 | 0.03 | 0.98 |
| ECOG PS (1 vs 0) (%) | 0.15 | 0.54 | 0.03 |
| FLIPI ≥3 (Yes) (%) | -0.71 | 0.61 | 0.25 |
| Ann Arbor Stage III/IV (Yes) (%) | 0.00 | 0.58 | 1.00 |
| Prior therapies ≥3 (%) | -1.23 | 2.40 | 0.61 |
| Refractory to last line (Yes) (%) | 4.73 | 2.63 | 0.07 |
| Refractory to any prior anti-CD20 mAb containing regimen (Yes) (%) | 0.99 | 0.74 | 0.18 |
| Double refractory (Yes) (%) | -0.61 | 0.56 | 0.28 |
| POD24 (Yes) (%) | -0.30 | 0.77 | 0.70 |
| Bone marrow involvement (Yes) (%) | -0.02 | 0.54 | 0.97 |
| Prior ASCT (Yes) (%) | 1.92 | 0.77 | 0.01 |
| Size of the largest node lesion [cm] (mean) | -0.07 | 0.08 | 0.38 |
| Low Hgb (Yes) (%) | 0.34 | 0.51 | 0.50 |
| High LDH (Yes) (%) | 0.92 | 0.54 | 0.09 |
| Time since completion of last therapy [months] (mean) | -0.01 | 0.01 | 0.33 |
| Age (mean)*Prior therapies ≥3 (%) | 0.01 | 0.04 | 0.77 |
| Age (mean)*Refractory to last line (Yes) (%) | -0.07 | 0.04 | 0.07 |
| Refractory to last line (Yes) (%)*POD24 (Yes) (%) | 0.19 | 0.99 | 0.85 |

Abbreviations: ASCT, autologous stem cell transplant; ECOG PS, Eastern Cooperative Oncology Group Performance Status; FLIPI, Follicular lymphoma international prognostic index; Hgb, hemoglobin; LDH, lactate dehydrogenase; POD24, Progression of disease within 24 months; SE, standard error.

**Table S2: Summary of model parameters for all regression adjustment models explored**

|  | **Base-case** | | | **Second lowest AIC model w/o convergence issues** | | | | **Third lowest AIC model w/o convergence issues** | | | | **Fourth lowest AIC model w/o convergence issues** | | | | **Regression adjustment (lowest AIC, assuming log-normal distribution of event times)** | | | | **Regression adjustment (lowest AIC, assuming log-logistic distribution of event times)** | | | | **Regression adjustment (lowest AIC, assuming exponential distribution of event times)** | | | |
| --- | --- | --- | --- | --- | --- | --- | --- | --- | --- | --- | --- | --- | --- | --- | --- | --- | --- | --- | --- | --- | --- | --- | --- | --- | --- | --- | --- |
| **PFS, rituximab + bendamustine** | | | | | | | | | | | | | | | | | | | | | | | | | | | |
| **Variable** | **Est.** | **SE** | **p** | | **Est.** | **SE** | **p** | | **Est.** | **SE** | **p** | | **Est.** | **SE** | **p** | | **Est.** | **SE** | **p** | | **Est.** | **SE** | **p** | | **Est.** | **SE** | **p** |
| (Intercept) | 3.86 | 0.30 | 0.00 | | 4.19 | 0.41 | 0.00 | | 3.79 | 0.30 | 0.00 | | 4.08 | 0.41 | 0.00 | | 3.65 | 0.30 | 0.00 | | 3.58 | 0.26 | 0.00 | | 4.01 | 0.41 | 0.00 |
| Refractory to any prior anti-CD20 mAb containing regimen (Yes) (%) | -0.57 | 0.33 | 0.08 | | -0.60 | -1.81 | 0.07 | | -0.73 | 0.33 | 0.03 | | -0.77 | 0.34 | 0.02 | | -1.00 | 0.34 | 0.00 | | -0.91 | 0.30 | 0.00 | | -0.95 | 0.46 | 0.04 |
| POD24 | -0.42 | 0.26 | 0.11 | | -0.45 | 0.26 | 0.09 | | NA | NA | NA | | NA | NA | NA | | NA | NA | NA | | NA | NA | NA | | NA | NA | NA |
| Size of the largest node lesion [cm] | NA | NA | NA | | -0.05 | 0.04 | 0.17 | | NA | NA | NA | | -0.05 | 0.04 | 0.23 | | NA | NA | NA | | NA | NA | NA | | NA | NA | NA |
| Log (scale) | -0.39 | 0.15 | 0.01 | | -0.41 | 0.15 | 0.01 | | -0.35 | 0.15 | 0.02 | | -0.37 | 0.15 | 0.02 | | -0.10 | 0.14 | 0.46 | | -0.72 | 0.16 | 0.00 | | NA | NA | NA |
| **PFS, mosunetuzumab** | | | | | | | | | | | | | | | | | | | | | | | | | | | |
| **Variable** | **Est.** | **SE** | **p** | | **Est.** | **SE** | **p** | | **Est.** | **SE** | **p** | | **Est.** | **SE** | **p** | | **Est.** | **SE** | **p** | | **Est.** | **SE** | **p** | | **Est.** | **SE** | **p** |
| (Intercept) | 4.08 | 0.69 | 0.00 | | 3.68 | 0.61 | 0.00 | | 2.86 | 0.31 | 0.00 | | 3.22 | 0.43 | 0.00 | | 2.82 | 0.42 | 0.00 | | 2.84 | 0.42 | 0.00 | | 4.15 | 0.74 | 0.00 |
| High LDH (Yes) (%) | -0.17 | 0.09 | 0.07 | | NA | NA | NA | | NA | NA | NA | | -0.69 | 0.50 | 0.17 | | -0.78 | 0.53 | 0.14 | | -0.78 | 0.53 | 0.14 | | -0.72 | 0.50 | 0.15 |
| Size of the largest node lesion [cm] | -0.68 | 0.47 | 0.15 | | -0.16 | 0.09 | 0.09 | | NA | NA | NA | | NA | NA | NA | | NA | NA | NA | | NA | NA | NA | | -0.17 | 0.10 | 0.09 |
| Time since completion of last therapy [months] (mean) | 0.06 | 0.03 | 0.04 | | 0.07 | 0.03 | 0.03 | | 0.07 | 0.03 | 0.03 | | 0.06 | 0.03 | 0.05 | | 0.06 | 0.03 | 0.02 | | 0.06 | 0.03 | 0.03 | | 0.06 | 0.03 | 0.03 |
| Log (scale) | -0.09 | 0.21 | 0.67 | | -0.06 | 0.21 | 0.76 | | 0.00 | 0.21 | 1.00 | | -0.02 | 0.21 | 0.94 | | 0.31 | 0.19 | 0.10 | | -0.23 | 0.21 | 0.27 | | NA | NA | NA |
| **OS, rituximab + bendamustine** | | | | | | | | | | | | | | | | | | | | | | | | | | | |
| **Variable** | **Est.** | **SE** | **p** | | **Est.** | **SE** | **p** | | **Est.** | **SE** | **p** | | **Est.** | **SE** | **p** | | **Est.** | **SE** | **p** | | **Est.** | **SE** | **p** | | **Est.** | **SE** | **p** |
| (Intercept) | 8.96 | 1.59 | 0.00 | | 10.09 | 2.02 | 0.00 | | 10.53 | 2.20 | 0.00 | | 15.28 | 2.79 | 0.00 | | 8.82 | 1.73 | 0.00 | | 9.08 | 1.75 | 0.00 | | 20.10 | 4.83 | 0.00 |
| Age (mean) | -0.04 | 0.02 | 0.05 | | -0.05 | 0.02 | 0.05 | | -0.05 | 0.03 | 0.06 | | -0.10 | 0.03 | 0.00 | | -0.05 | 0.02 | 0.02 | | -0.05 | 0.02 | 0.05 | | -0.14 | 0.05 | 0.01 |
| ECOG PS (1 vs 0) (%) | -0.87 | 0.45 | 0.06 | | -0.72 | 0.48 | 0.14 | | NA | NA | NA | | NA | NA | NA | | -1.30 | 0.55 | 0.02 | | -0.88 | 0.53 | 0.10 | | NA | NA | NA |
| Prior therapies ≥3 (%) | NA | NA | NA | | NA | NA | NA | | NA | NA | NA | | -1.36 | 0.60 | 0.02 | | NA | NA | NA | | NA | NA | NA | | -2.35 | 1.14 | 0.04 |
| Refractory to last line (Yes) (%) | -1.15 | 0.39 | 0.00 | | -1.25 | 0.44 | 0.00 | | -1.59 | 0.45 | 0.00 | | -1.59 | 0.45 | 0.00 | | -1.05 | 0.44 | 0.02 | | -1.06 | 0.46 | 0.02 | | -2.07 | 0.90 | 0.02 |
| Size of the largest node lesion [cm] | NA | NA | NA | | -0.10 | 0.07 | 0.18 | | -0.13 | 0.07 | 0.06 | | -0.23 | 0.09 | 0.01 | | NA | NA | NA | | NA | NA | NA | | -0.32 | 0.16 | 0.05 |
| Low Hgb (Yes) (%) | -0.94 | 0.45 | 0.04 | | -0.85 | 0.51 | 0.10 | | -1.28 | 0.45 | 0.00 | | NA | NA | NA | | -0.91 | 0.43 | 0.04 | | -0.88 | 0.49 | 0.07 | | NA | NA | NA |
| High LDH (Yes) (%) | -1.59 | 0.44 | 0.00 | | -1.63 | 0.41 | 0.00 | | -1.38 | 0.41 | 0.00 | | -1.42 | 0.43 | 0.00 | | -2.22 | 0.68 | 0.00 | | -1.56 | 0.55 | 0.00 | | NA | NA | NA |
| Log (scale) | -0.90 | 0.30 | 0.00 | | -0.92 | 0.29 | 0.00 | | -0.85 | 0.30 | 0.00 | | -0.75 | 0.31 | 0.01 | | -0.40 | 0.27 | 0.14 | | -0.94 | 0.31 | 0.00 | | -1.88 | 0.86 | 0.03 |
| **OS, mosunetuzumab** | | | | | | | | | | | | | | | | | | | | | | | | | | | |
| **Variable** | **Est.** | **SE** | **p** | | **Est.** | **SE** | **p** | | **Est.** | **SE** | **p** | | **Est.** | **SE** | **p** | | **Est.** | **SE** | **p** | | **Est.** | **SE** | **p** | | **Est.** | **SE** | **p** |
| (Intercept) | 9.75 | 4.02 | 0.02 | | 13.98 | 6.62 | 0.03 | | 9.34 | 3.80 | 0.01 | | 10.25 | 4.50 | 0.02 | | 11.86 | 5.36 | 0.03 | | 10.44 | 4.74 | 0.03 | | 11.82 | 3.48 | 0.00 |
| Age |  |  |  | | -0.05 | 0.05 | 0.26 | | -1.76 | 1.56 | 0.26 | | NA | NA | NA | | NA | NA | NA | | NA | NA | NA | | NA | NA | NA |
| FLIPI ≥3 (Yes) (%) | -2.00 | 1.62 | 0.22 | | -2.36 | 1.70 | 0.16 | | -0.47 | 0.26 | 0.07 | | -1.87 | 1.71 | 0.27 | | -3.14 | 2.55 | 0.22 | | -2.62 | 2.15 | 0.22 | | NA | NA | NA |
| Size of the largest node lesion [cm] (mean) | -0.47 | 0.27 | 0.08 | | -0.55 | 0.30 | 0.07 | | 0.03 | 0.05 | 0.61 | | -0.51 | 0.30 | 0.09 | | -0.61 | 0.35 | 0.09 | | -0.52 | 0.31 | 0.10 | | -0.61 | 0.23 | 0.01 |
| High LDH (Yes) (%) | NA | NA | NA | | NA | NA | NA | | NA | NA | NA | | -0.63 | 0.96 | 0.51 | | NA | NA | NA | | NA | NA | NA | | NA | NA | NA |
| Log (scale) | -0.31 | 0.48 | 0.52 | | -0.42 | 0.48 | 0.38 | | -0.29 | 0.48 | 0.55 | | NA | NA | NA | | 0.38 | 0.41 | 0.35 | | -0.41 | 0.47 | 0.39 | | NA | NA | NA |

Abbreviations: AIC, Akaike information criterion; ECOG PS, Eastern Cooperative Oncology Group performance status; est., estimate; FLIPI, Follicular Lumphoma International Prognostic Index; Hgb, hemoglobin; LDH, lactate dehydrogenase; mAb, monoclonal antibody; OS, overall survival; p, p-value; PFS, progression-free survival; SE, standard error.

**Table S3: Comparison of results for mosunetuzumab versus BR across methods, HR**

| Method for estimating HR | HR (95% CI) | |
| --- | --- | --- |
|  | **OS** | **PFS** |
| Unadjusted | 0.94 (0.31, 2.81) | 0.90 (0.54, 1.50) |
| Optimal pair matching plus covariate adjustment | 0.53 (0.03, 2.85) | 0.70 (0.29, 1.31) |
| Matching plus regression adjustment (reference case) | 0.30 (0.05, 5.28) | 0.43 (0.13, 0.91) |

Abbreviations: BR, rituximab plus bendamustine; CI, confidence interval; HR, hazard ratio; ITC, indirect treatment comparison; OS, overall survival.
HRs presented for the comparison of mosunetuzumab versus rituximab plus bendamustine. HRs <1 favor mosunetuzumab.

**Table S4: Comparison of results for mosunetuzumab versus BR across methods, RMST difference**

| Method for estimating RMST difference | RMST difference (95% CI) | |
| --- | --- | --- |
|  | **OS** | **PFS** |
| Unadjusted | 0.30 (-2.51, 3.11) | 0.003 (-3.61, 3.61) |
| Optimal pair matching plus covariate adjustment | 0.78 (-2.05, 3.68) | 1.26 (-2.81, 4.98) |
| Matching plus regression adjustment (reference case) | 5.00 (-5.40, 11.56) | 7.57 (-0.29, 14.70) |

Abbreviations: BR, rituximab plus bendamustine; CI, confidence interval; ITC, indirect treatment comparison; OS, overall survival; RMST, restricted mean survival time.
RMST differences presented for the comparison of mosunetuzumab versus rituximab plus bendamustine. RMST difference >1 favor mosunetuzumab.

**Table S5: Summary of survival rates at 2 and 3 years, median survivals, and RMST**

|  | Mosunetuzumab (95% CI) | | | | BR (95% CI) | | | |
| --- | --- | --- | --- | --- | --- | --- | --- | --- |
| **OS** | | | | | | | | |
|  | **2-year survival rate, %** | **3-year survival rate, %** | **Median survival, months** | **RMST, months** | **2-year survival rate, %** | **3-year survival rate, %** | **Median survival, months** | **RMST, months** |
| Reference case (lowest AIC and assuming Weibull distribution of event times) | 0.93 (0.45, 0.99) | 0.93 (0.68, 0.98) | - | 47.74 (24.83, 50.09) | 0.88 (0.71, 0.97) | 0.88 (0.58, 0.96) | - | 42.73 (29.12, 49.15) |
| Regression adjustment (second lowest AIC model* and assuming Weibull distribution of event times) | 0.92 (0.49, 0.98) | 0.92 (0.66, 0.98) | - | 47.45 (24.65, 50.01) | 0.89 (0.72, 0.96) | 0.89 (0.72, 0.96) | - | 43.00 (29.23, 48.64) |
| Regression adjustment (third lowest AIC model* and assuming Weibull distribution of event times)) | 0.94 (0.47, 0.99) | 0.94 (0.69, 0.99) | - | 48.69 (24.30, 50.46) | 0.88 (0.74, 0.97) | 0.88 (0.59, 0.94) | - | 42.86 (29.29, 48.84) |
| Regression adjustment (fourth lowest AIC model* and assuming Weibull distribution of event times)) | 0.93 (0.48, 0.98) | 0.93 (0.73, 0.98) | - | 47.79 (24.35, 50.02) | 0.87 (0.76, 0.97) | 0.87 (0.67, 0.94) | - | 43.76 (29.16, 48.76) |
| Regression adjustment (lowest AIC, assuming log-normal distribution of event times) | 0.94 (0.87, 0.99) | 0.94 (0.75, 0.99) | - | 48.70 (43.27, 50.50) | 0.88 (0.72, 0.97) | 0.88 (0.63, 0.95) | - | 44.55 (37.00, 48.90) |
| Regression adjustment (lowest AIC, assuming log-logistic distribution of event times) | 0.94 (0.87, 0.99) | 0.94 (0.75, 0.99) | - | 48.64 (42.21, 50.50) | 0.91 (0.75, 0.97) | 0.91 (0.64, 0.96) | - | 45.35 (38.41, 49.33) |
| Regression adjustment (lowest AIC, assuming exponential distribution of event times) | 0.93 (0.60, 0.99) | 0.93 (0.61, 0.99) | - | 48.16 (30.46, 50.51) | 0.89 (0.81, 0.97) | 0.89 (0.77, 0.95) | - | 45.97 (42.17, 49.06) |
| **PFS** | | | | | | | | |
|  | **2-year survival rate** | **3-year survival rate** | **Median survival** | **RMST** | **2-year survival rate** | **3-year survival rate** | **Median survival** | **RMST** |
| Reference case (lowest AIC and assuming Weibull distribution of event times) | 0.70 (0.44, 0.86) | 0.55 (0.29, 0.82) | 40.04 (20.66, 41.02) | 30.42 (23.94, 36.81) | 0.45 (0.19, 0.68) | 0.19 (0.05, 0.45) | 18.86 (13.34, 32.65) | 22.84 (17.95, 28.55) |
| Regression adjustment (second lowest AIC model* and assuming Weibull distribution of event times) | 0.71 (0.44, 0.86) | 0.57 (0.28, 0.84) | 38.44 (20.71, 41.17) | 30.78 (24.13, 36.88) | 0.40 (0.20, 0.64) | 0.19 (0.06, 0.42) | 18.86 (13.52, 30.27) | 23.19 (18.13, 28.61) |
| Regression adjustment (third lowest AIC model* and assuming Weibull distribution of event times)) | 0.71 (0.44, 0.87) | 0.51 (0.30, 0.86) | 37.41 (19.94, 41.01) | 30.39 (24.37, 37.20) | 0.30 (0.20, 0.77) | 0.27 (0.04, 0.35) | 19.83 (13.90, 29.48) | 23.85 (18.14, 28.65) |
| Regression adjustment (fourth lowest AIC model* and assuming Weibull distribution of event times)) | 0.74 (0.45, 0.87) | 0.53 (0.32, 0.84) | 37.36 (20.80, 41.26) | 30.84 (24.50, 36.83) | 0.30 (0.20, 0.72) | 0.27 (0.05, 0.37) | 21.03 (14.22, 31.72) | 23.96 (18.33, 29.15) |
| Regression adjustment (lowest AIC, assuming log-normal distribution of event times) | 0.79 (0.53, 0.87) | 0.74 (0.38, 0.87) | - | 34.22 (26.22, 37.61) | 0.31 (0.20, 0.77) | 0.26 (0.09, 0.67) | 22.20 (14.61, 35.96) | 24.89 (19.24, 32.73) |
| Regression adjustment (lowest AIC, assuming log-logistic distribution of event times) | 0.79 (0.56, 0.88) | 0.79 (0.42, 0.87) | - | 34.82 (27.31, 37.65) | 0.48 (0.21, 0.77) | 0.24 (0.09, 0.70) | 23.53 (15.42, 37.76) | 25.25 (19.65, 33.51) |
| Regression adjustment (lowest AIC, assuming exponential distribution of event times) | 0.71 (0.51, 0.86) | 0.57 (0.36, 0.82) | - | 31.16 (25.75, 36.57) | 0.31 (0.20, 0.77) | 0.27 (0.12, 0.35) | 21.80 (14.56, 33.67) | 24.69 (19.44, 30.74) |

Abbreviations: AIC, Akaike information criterion; BR, rituximab plus bendamustine; CI, confidence interval; ITC, indirect treatment comparison; PFS, progression-free survival; OS, overall survival; RMST, restricted mean survival time.
Where values are not presented for median survival, estimation was not possible as OS/PFS was not reached.

## Figures

Figure S1: Propensity score distribution


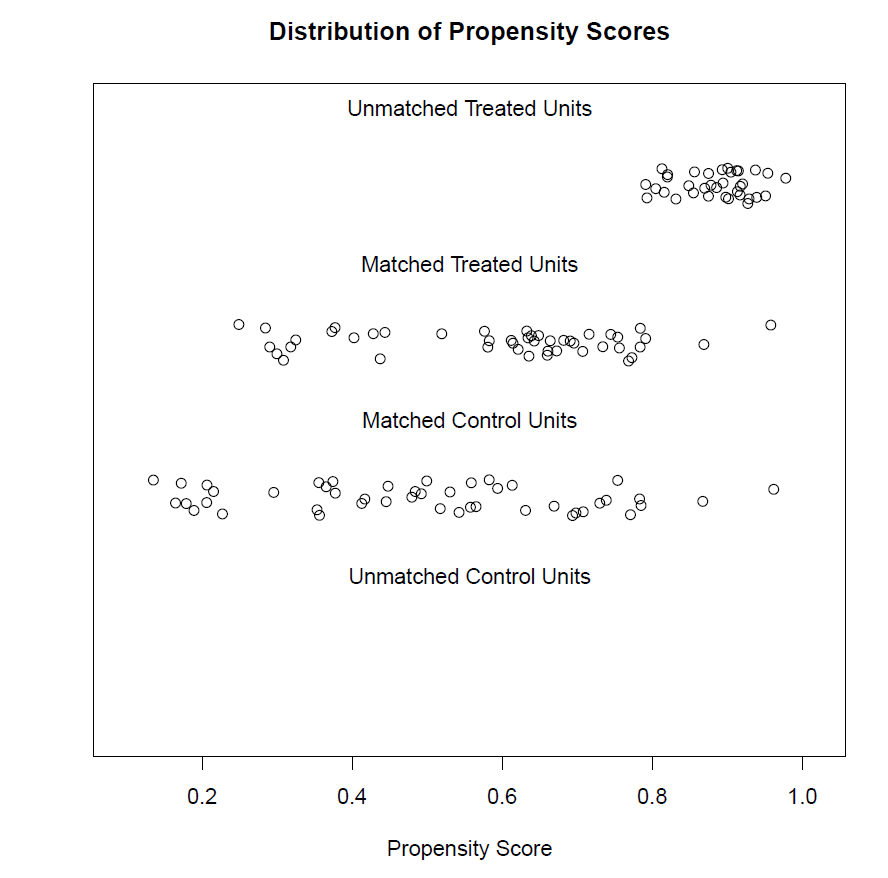


Figure S2: Love plots for the comparison with rituximab plus bendamustine coefficients using optimal pair matching and a propensity score model without (A) and with (B) interaction terms

A


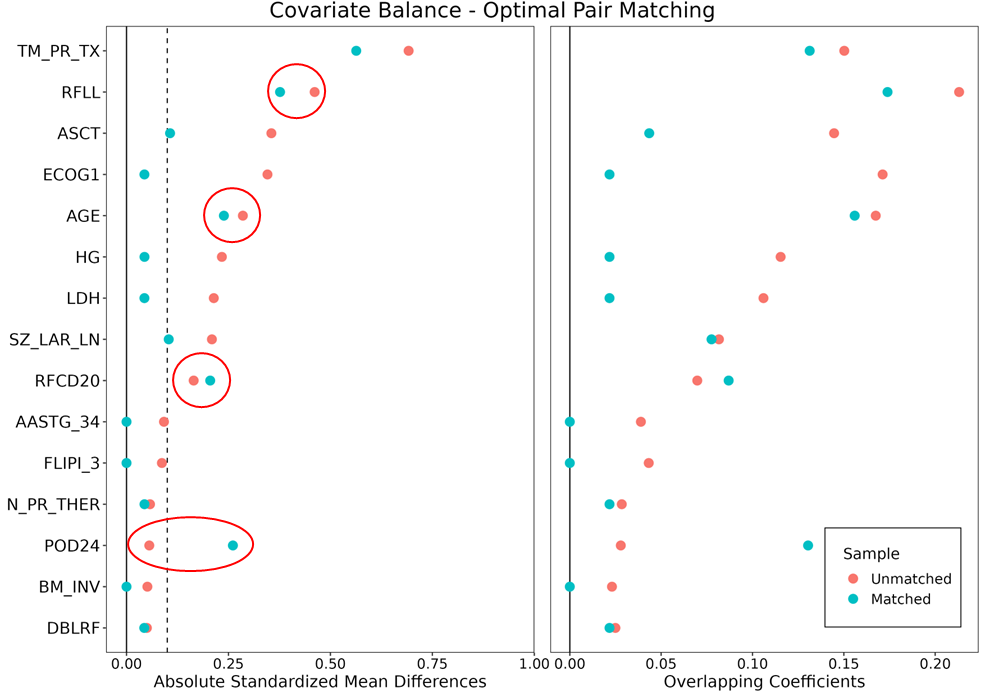


B


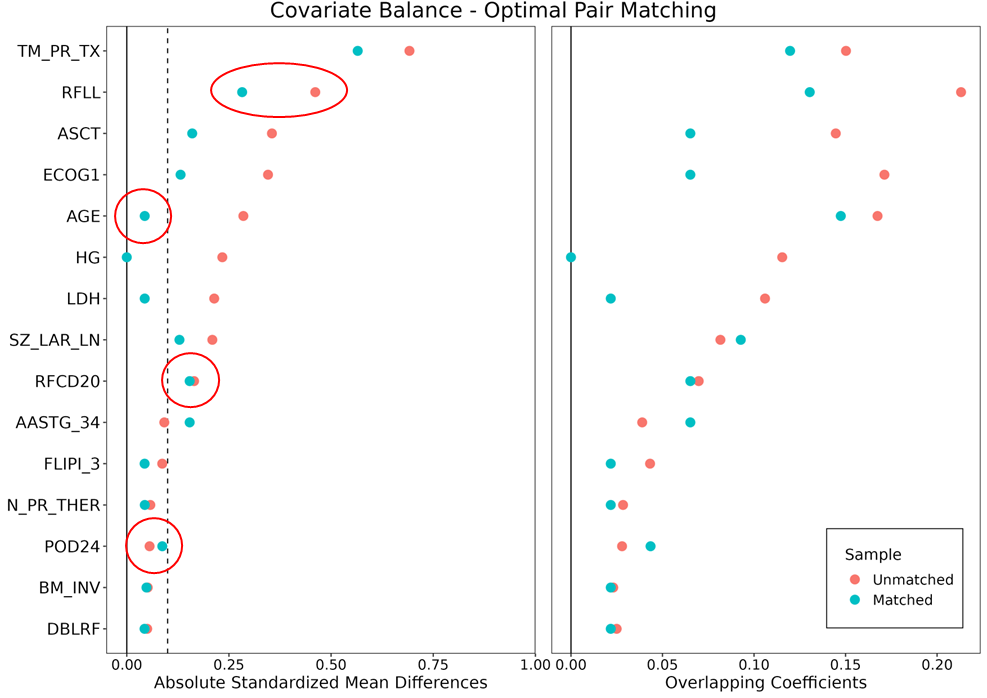


Legend: Total absolute standardised mean difference (aSMD) using the propensity score model without interactions = 2.07. Total aSMD using the propensity score model with interactions = 1.91. Sum of overlapping coefficients was similar in both scenarios (~0.91).

**Figure S3: Love plots for the comparison with rituximab plus bendamustine**

**A. NN matching**

**
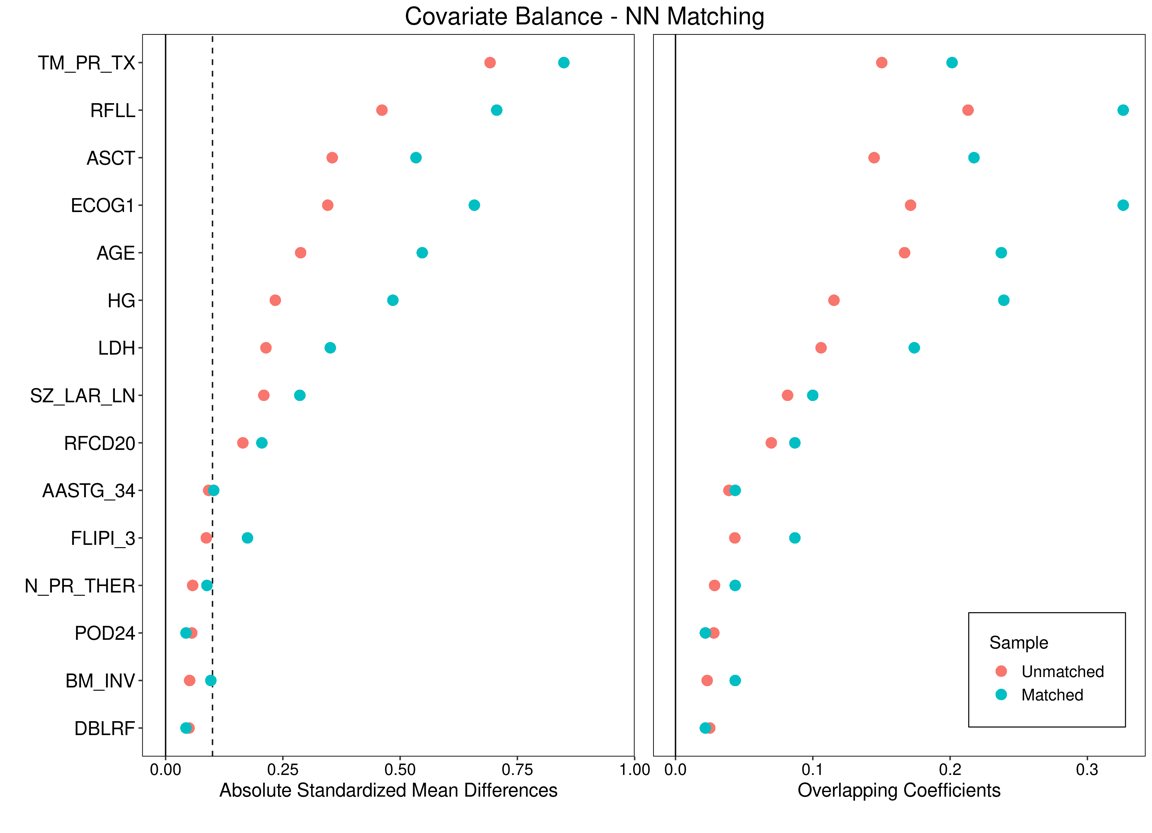
**

**B. Optimal matching**

**
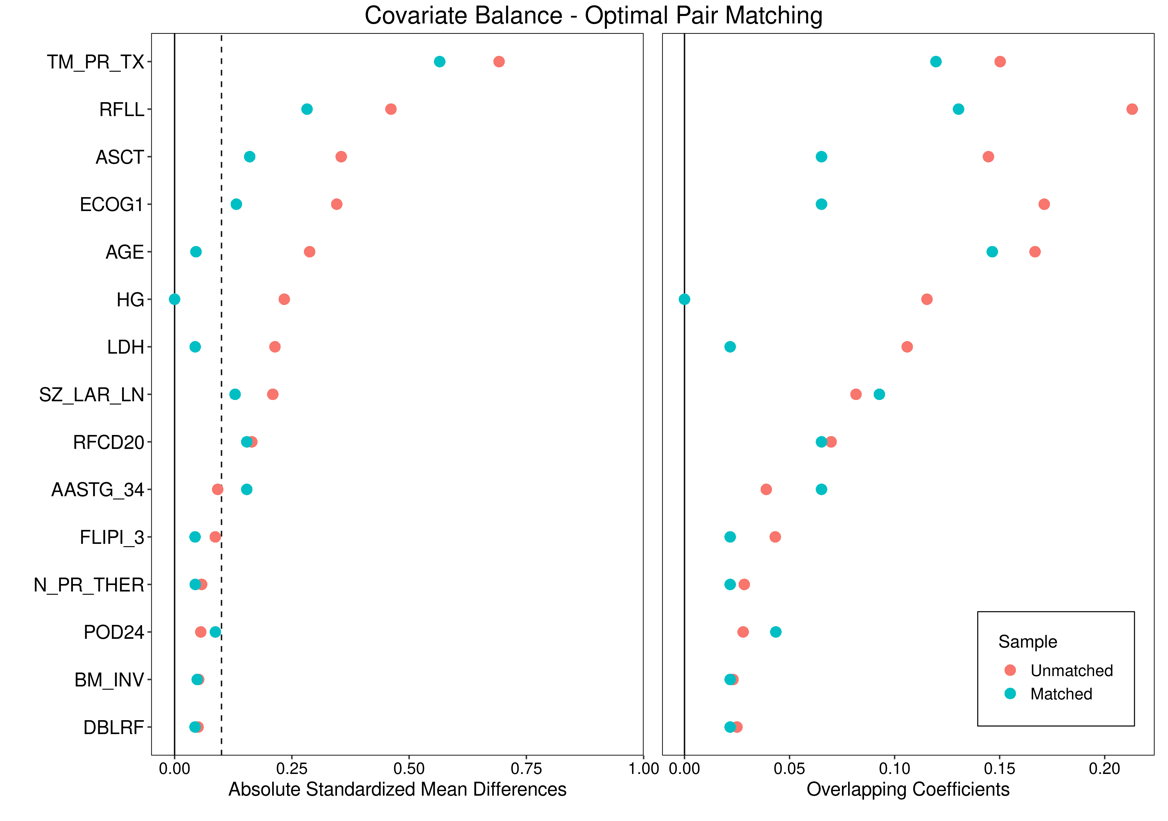
**

**C. Genetic matching**

**
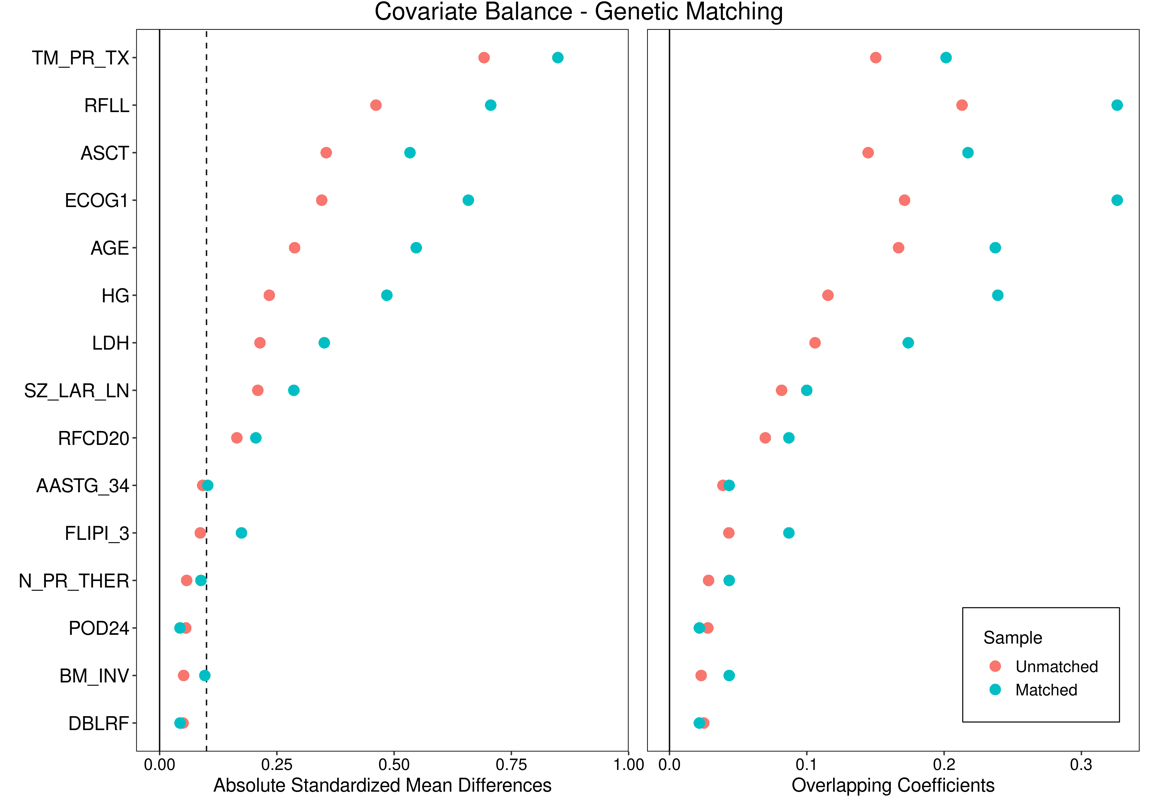
**

**D. NN matching with replacement**

**
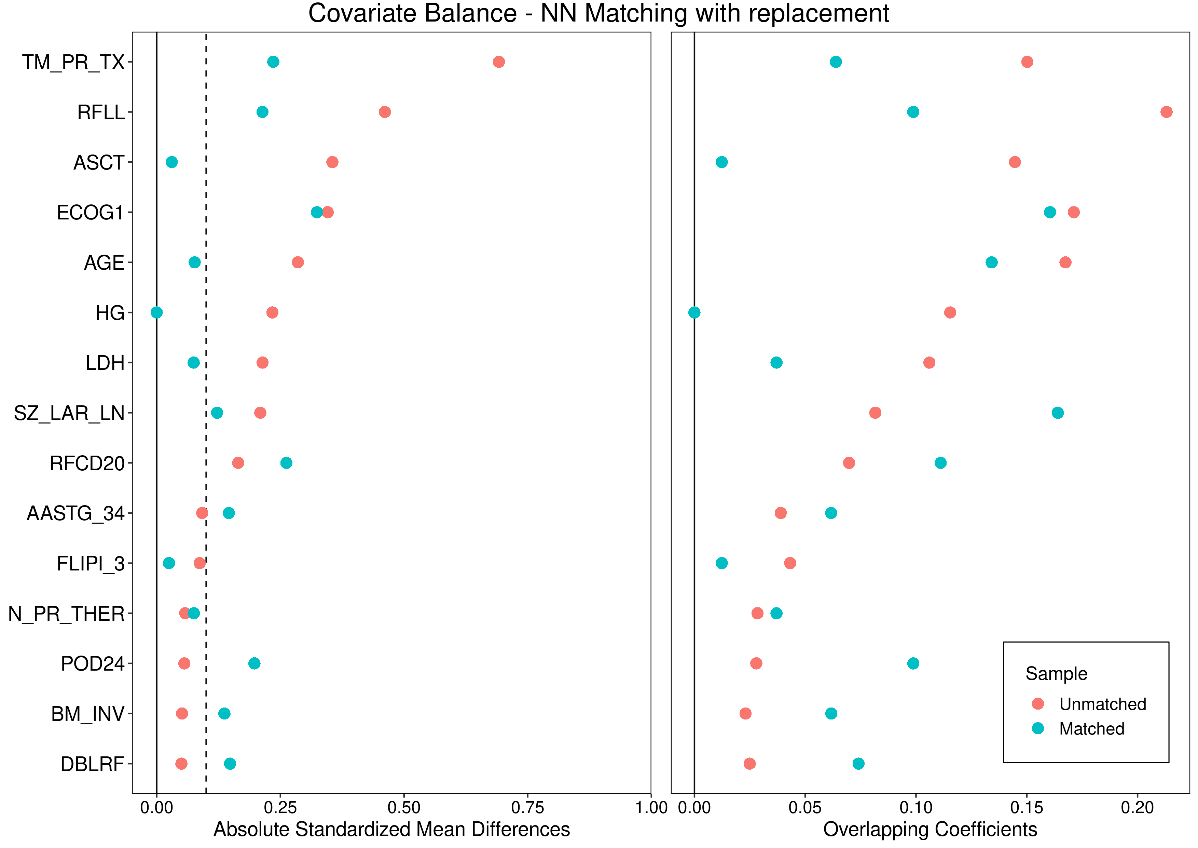
**

**E. Full matching**

**
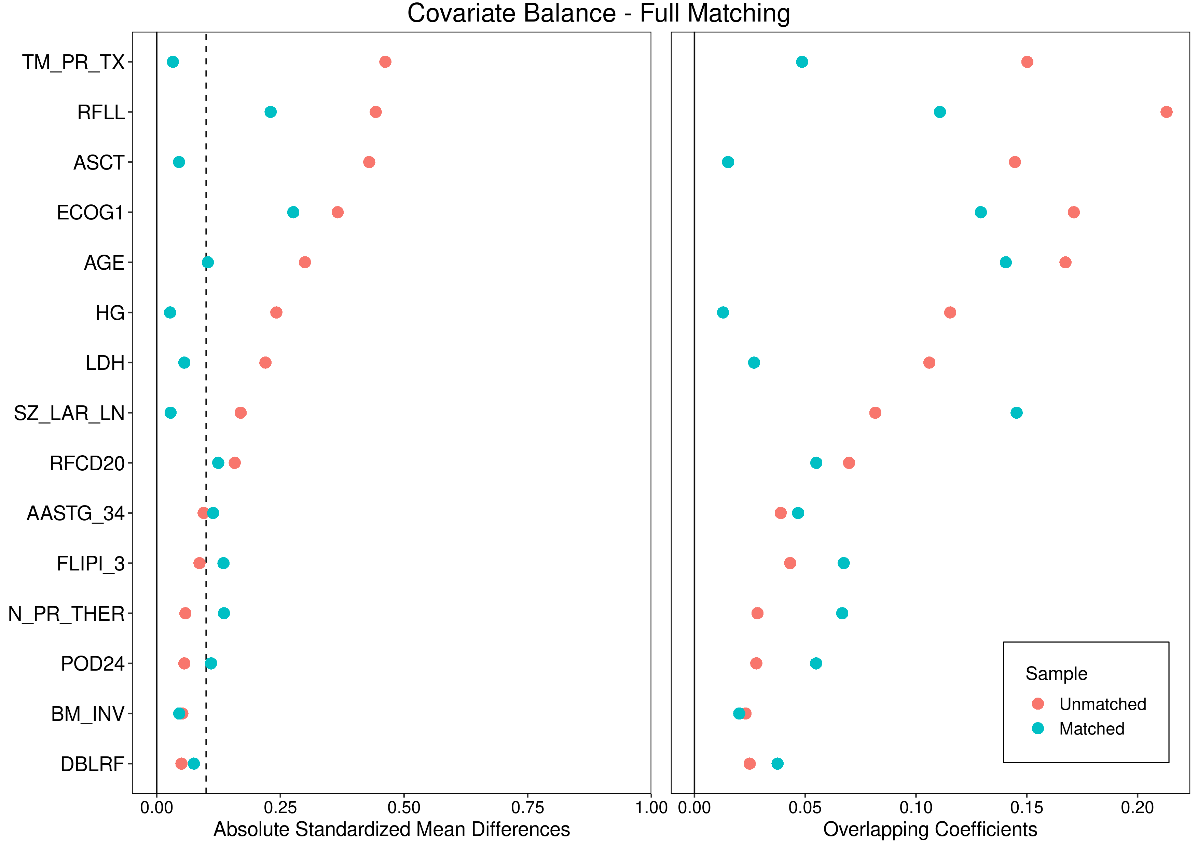
**

**Figure S4: KM plots of OS for all matching plus regression adjustment samples explored**

**KM plots of OS**

**A. ‘Doubly robust’ regression adjustment**

**
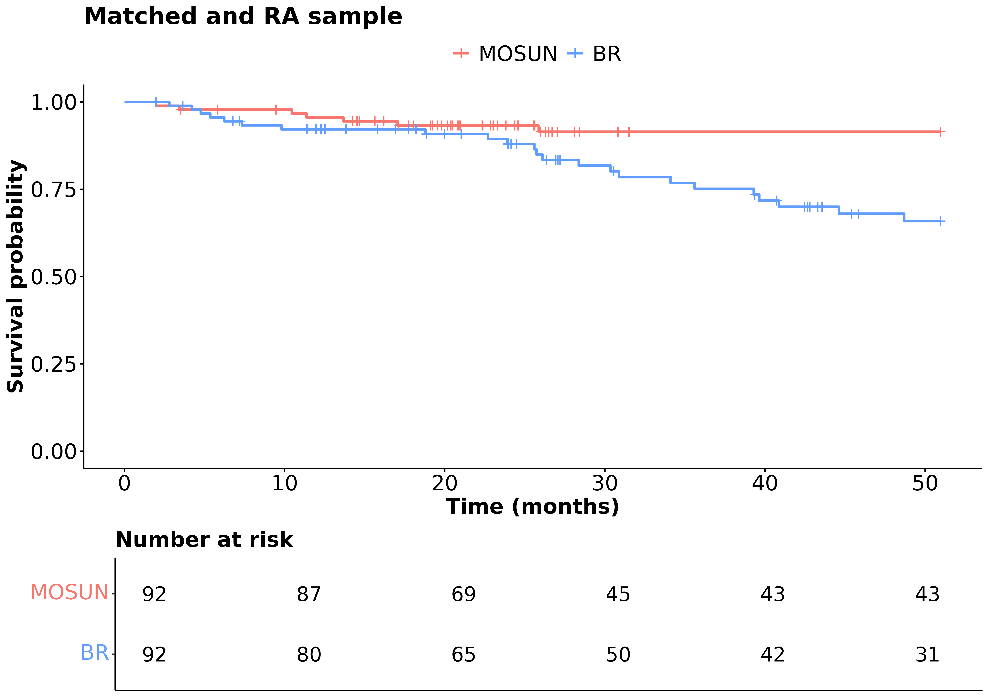
**

**B. ‘Doubly robust’ regression adjustment (using second lowest AIC model w/o convergence issues)**

**
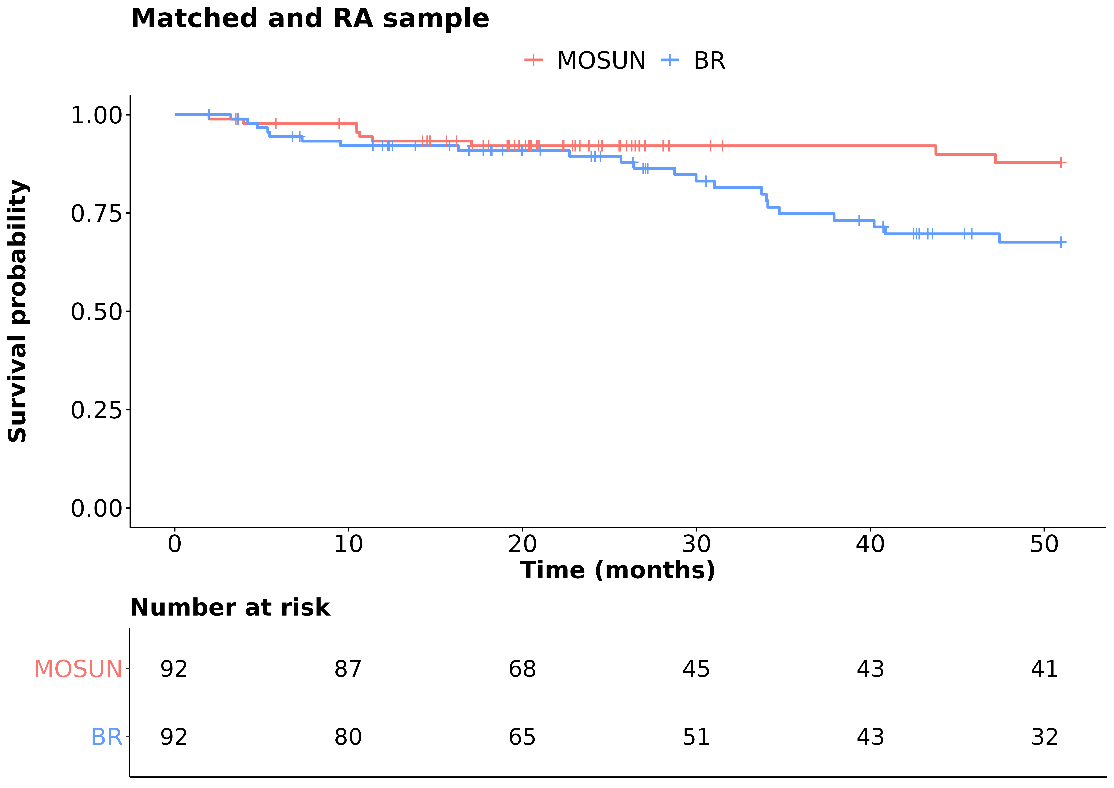
**

**C. Doubly robust’ regression adjustment (using third lowest AIC model w/o convergence issues)**

**
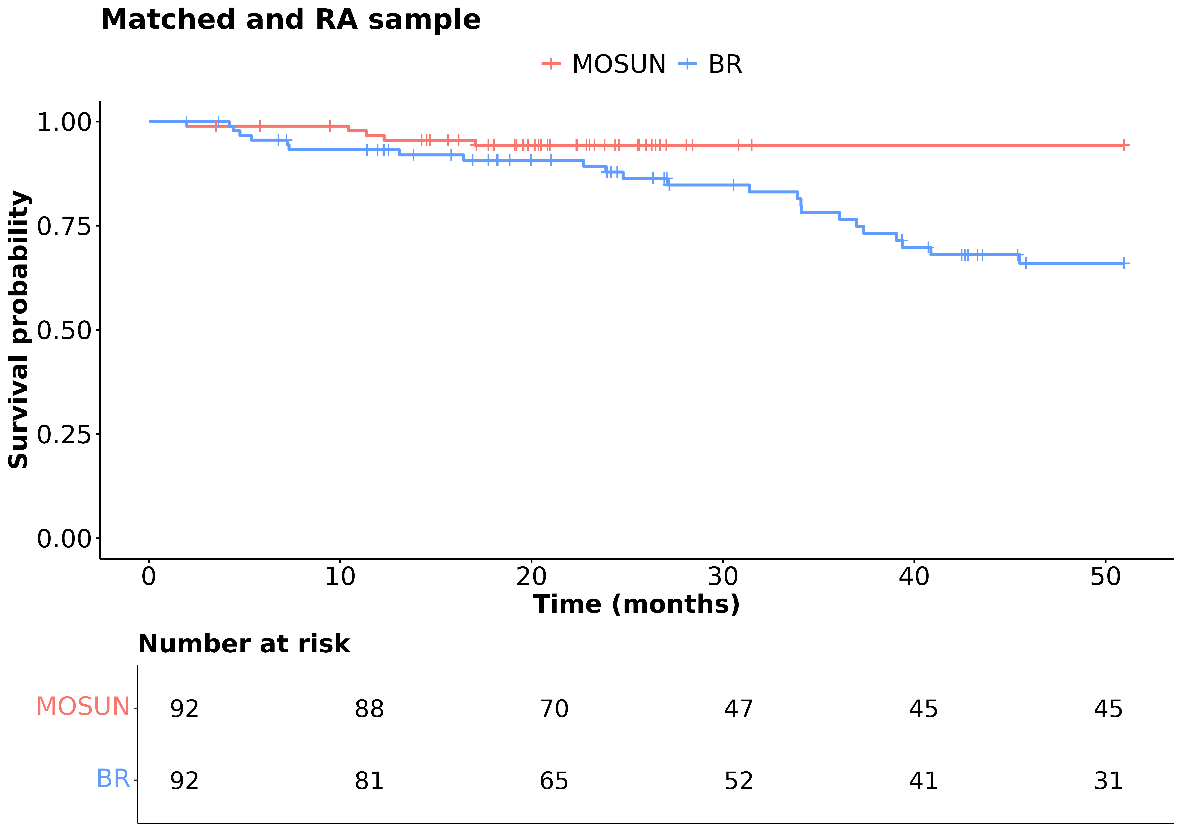
**

**D. ‘Doubly robust’ regression adjustment (using fourth lowest AIC model w/o convergence issues)**

**
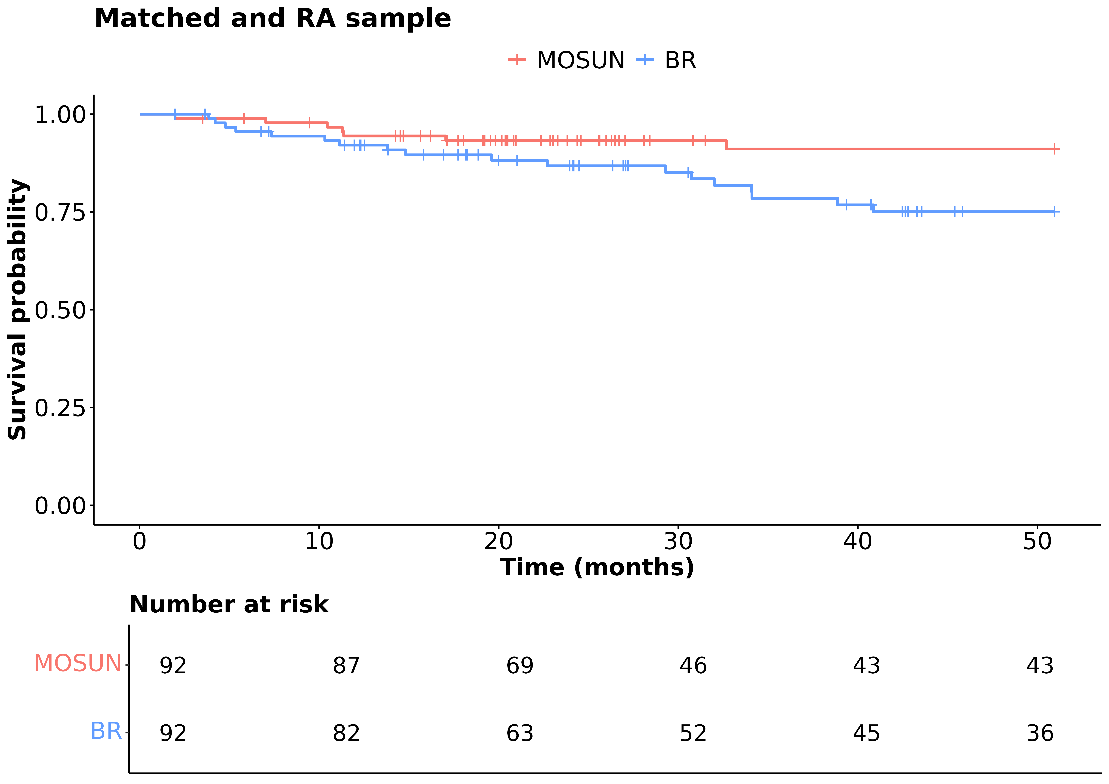
**

**E. ‘Doubly robust’ regression adjustment (Assuming log-normal distribution of event times)**

**
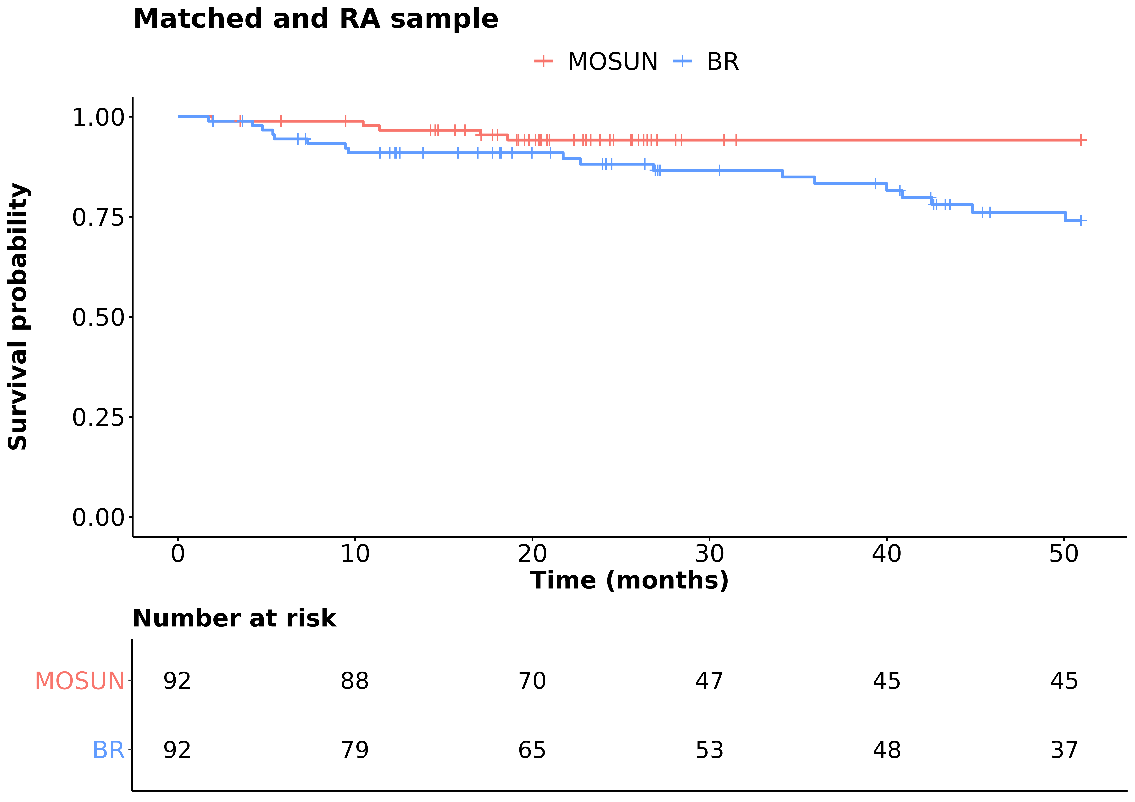
**

**F. ‘Doubly robust’ regression adjustment (Assuming log-logistic distribution of event times)**

**
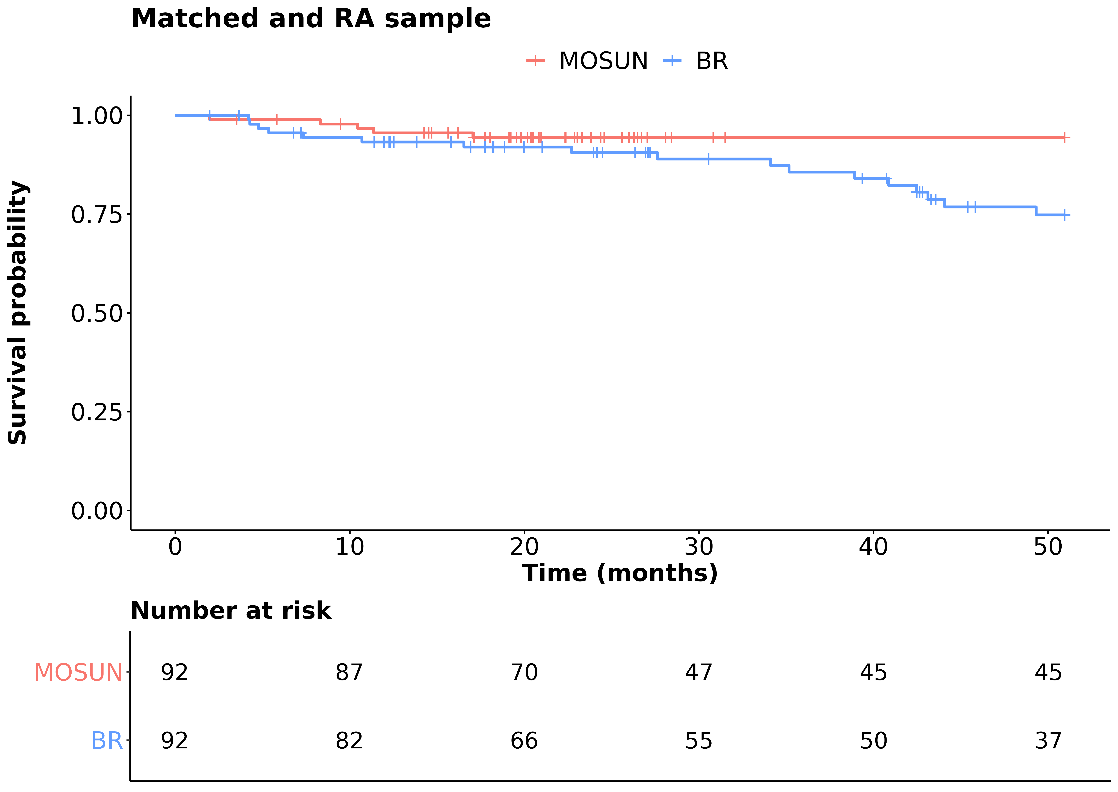
**

**G. ‘Doubly robust’ regression adjustment (Assuming exponential distribution of event times)**

**
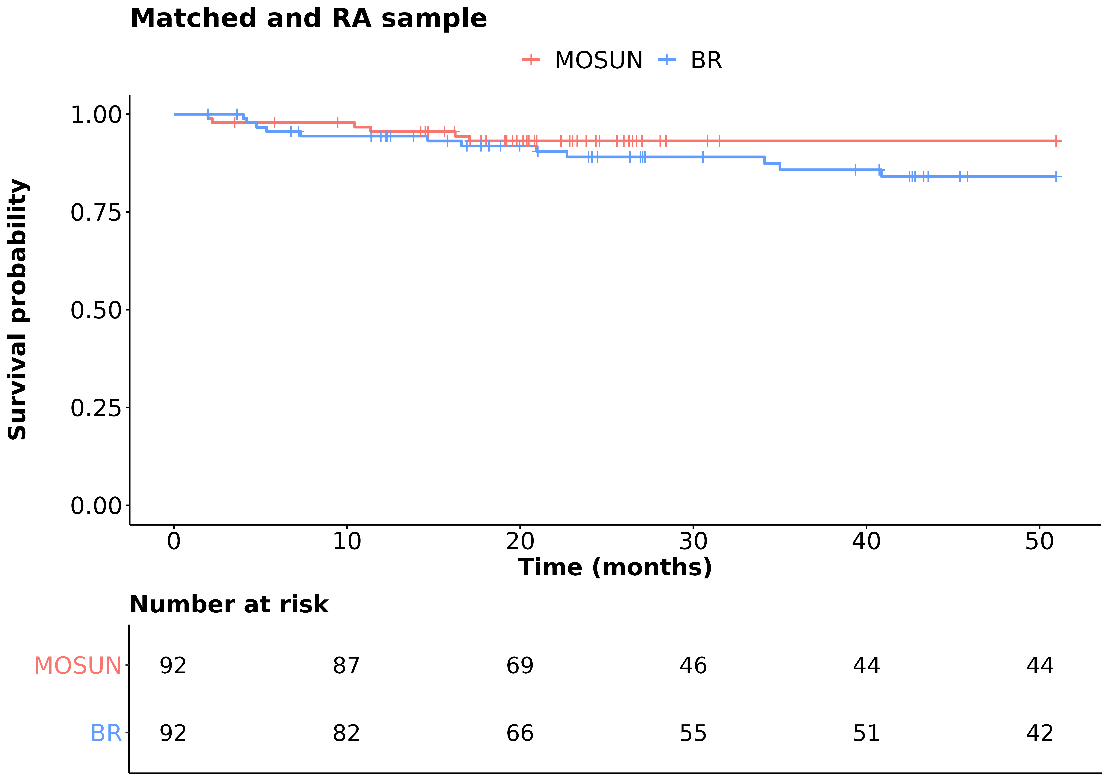
**

**KM plots of PFS**

**H. ‘Doubly robust’ regression adjustment**

**
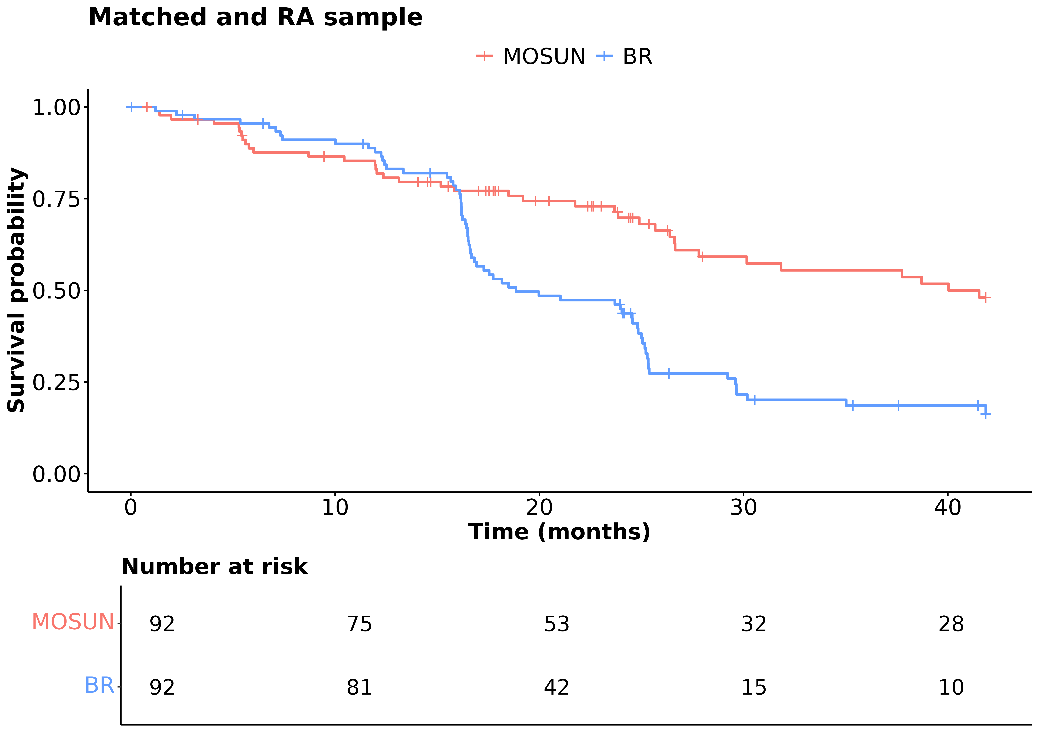
**

**I. ‘Doubly robust’ regression adjustment (using second lowest AIC model w/o convergence issues)**

**
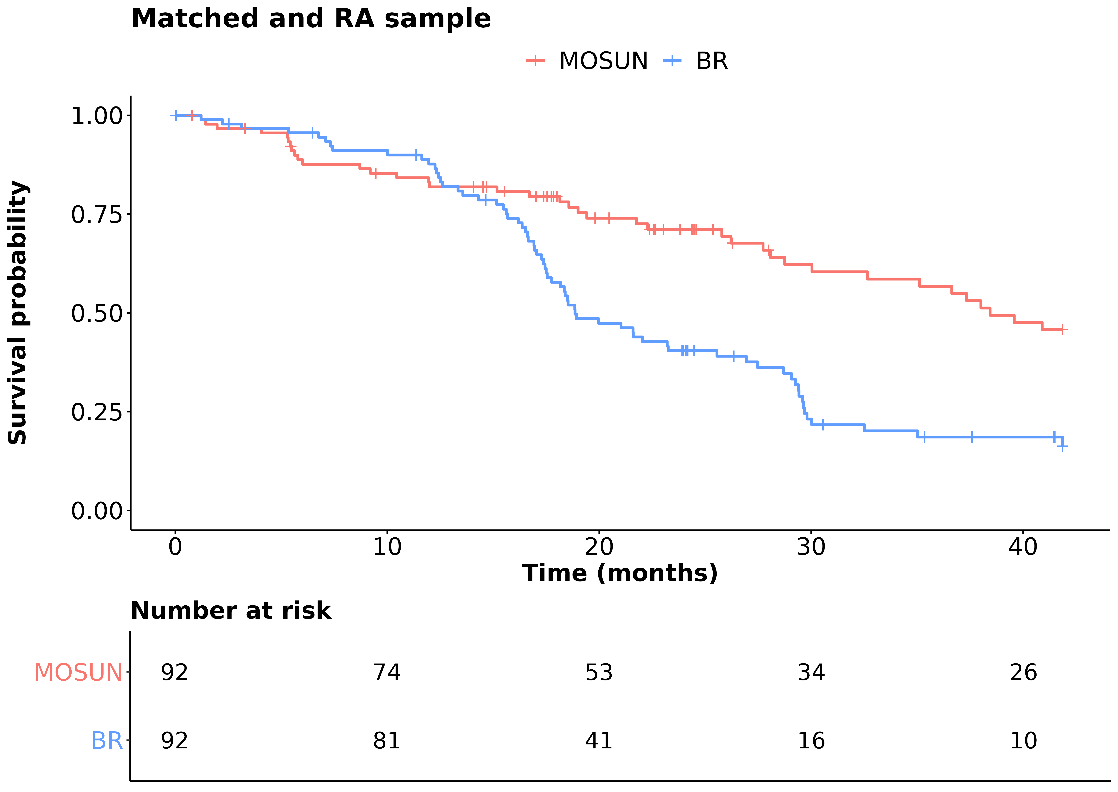
**

**J. ‘Doubly robust’ regression adjustment (using third lowest AIC model w/o convergence issues)**

**
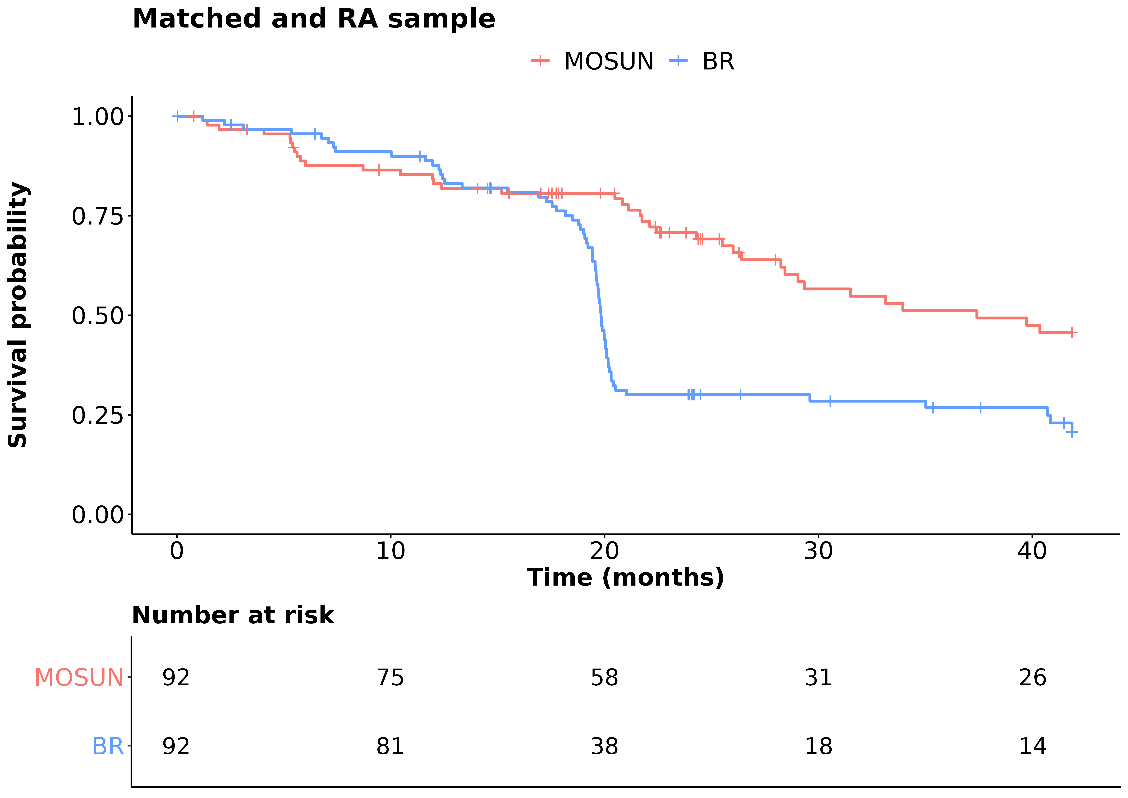
**

**K. ‘Doubly robust’ regression adjustment (using fourth lowest AIC model w/o convergence issues)**

**
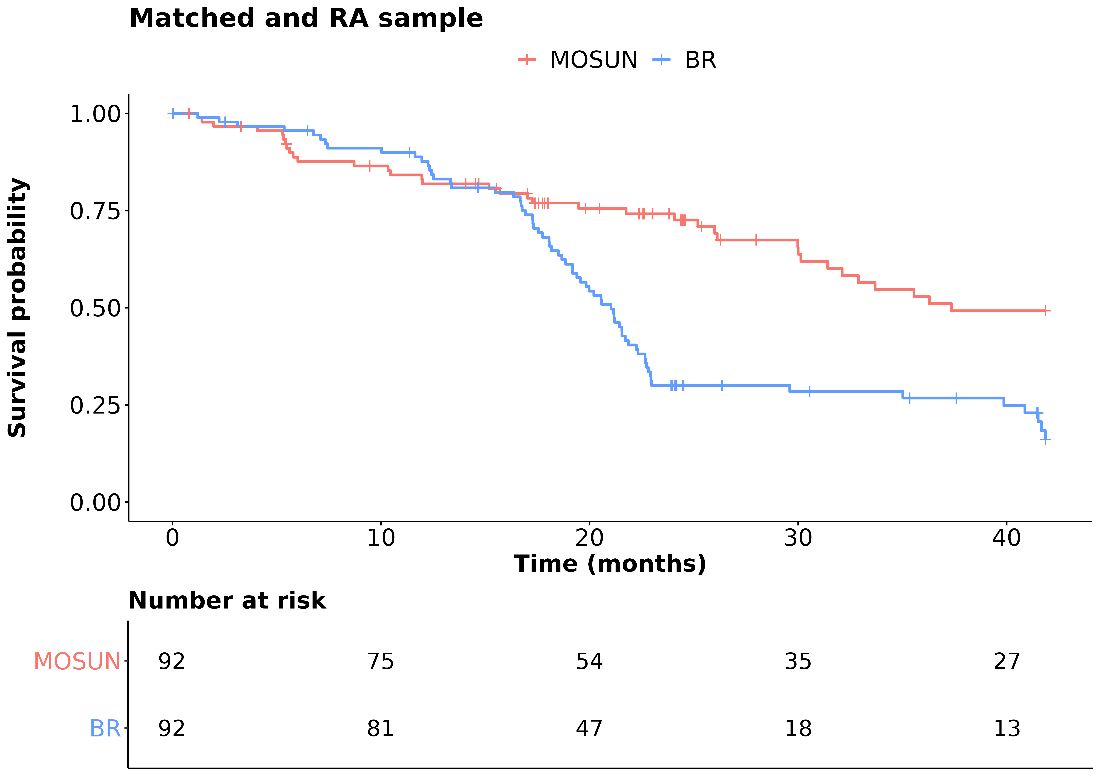
**

**L. ‘Doubly robust’ regression adjustment (Assuming log-normal distribution of event times)**

**
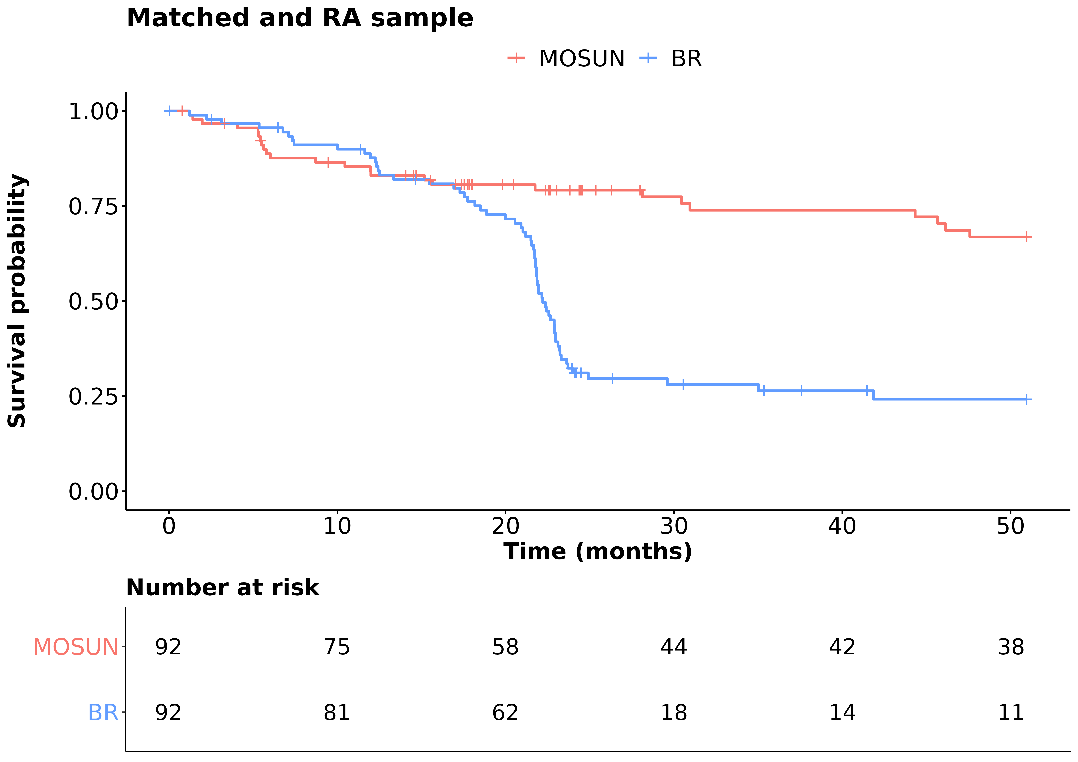
**

**M. ‘Doubly robust’ regression adjustment (Assuming log-logistic distribution of event times)**

**
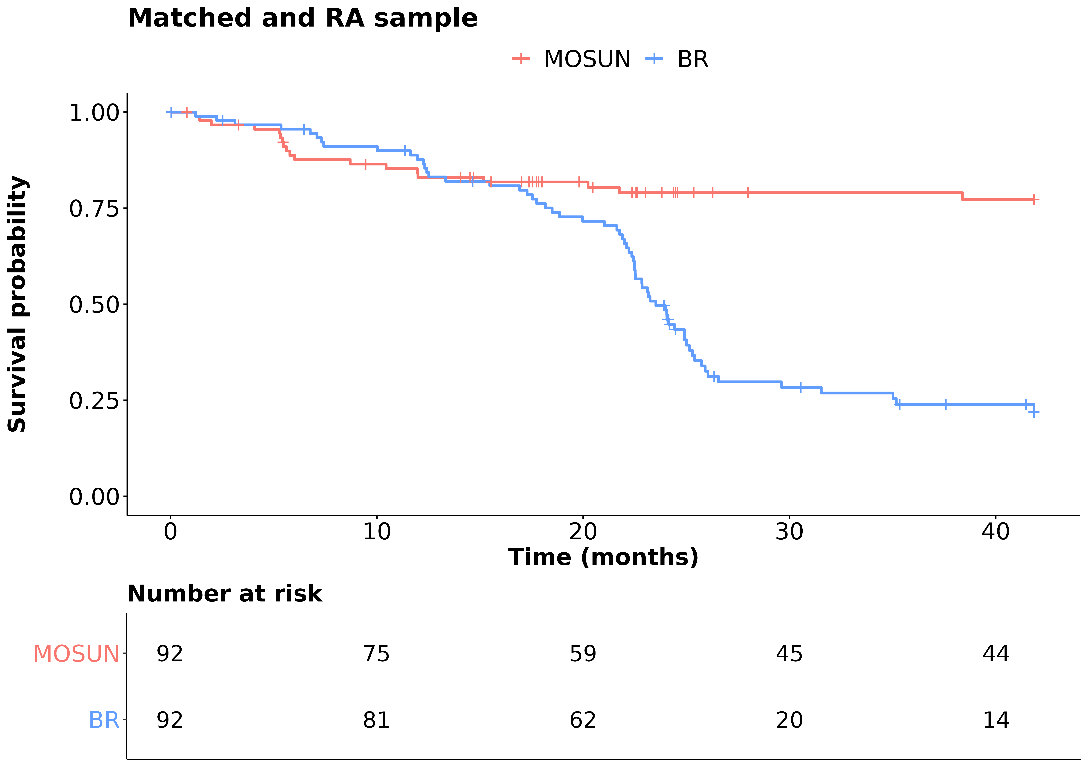
**

**N. ‘Doubly robust’ regression adjustment (Assuming exponential distribution of event times)**

**
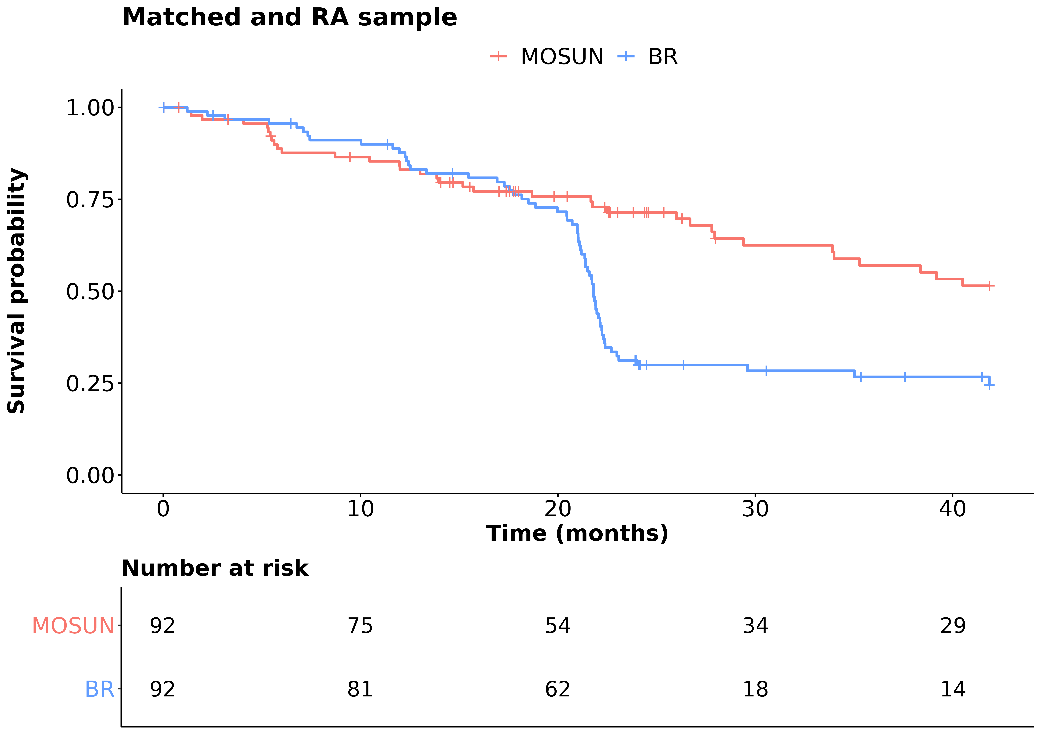
**

## R-code for analyses
